# Weak-solvation dilution for interphase preservation and extreme low-temperature sodium-ion storage

**DOI:** 10.1093/nsr/nwaf510

**Published:** 2025-11-17

**Authors:** Jinyu Yang, Mingxu Wang, Haoran Ji, Ziyue Li, Fengmei Wang, Zihao Zhang, Xinjie Li, Yanru Yang, Qin Li, Jiafeng Ruan, Fang Fang, Dalin Sun, Fei Wang

**Affiliations:** College of Smart Materials and Future Energy, Fudan University, Shanghai 200433, China; Yiwu Research Institute of Fudan University, Yiwu 322000, China; College of Smart Materials and Future Energy, Fudan University, Shanghai 200433, China; College of Smart Materials and Future Energy, Fudan University, Shanghai 200433, China; College of Smart Materials and Future Energy, Fudan University, Shanghai 200433, China; College of Smart Materials and Future Energy, Fudan University, Shanghai 200433, China; College of Smart Materials and Future Energy, Fudan University, Shanghai 200433, China; College of Smart Materials and Future Energy, Fudan University, Shanghai 200433, China; College of Smart Materials and Future Energy, Fudan University, Shanghai 200433, China; College of Smart Materials and Future Energy, Fudan University, Shanghai 200433, China; College of Smart Materials and Future Energy, Fudan University, Shanghai 200433, China; Inorganic Chemistry I, Technische Universität Dresden, Dresden 01069, Germany; College of Smart Materials and Future Energy, Fudan University, Shanghai 200433, China; Yiwu Research Institute of Fudan University, Yiwu 322000, China; School of Materials Science and Engineering, Anhui University, Hefei 230601, China; College of Smart Materials and Future Energy, Fudan University, Shanghai 200433, China; College of Smart Materials and Future Energy, Fudan University, Shanghai 200433, China; School of Materials Science and Engineering, Anhui University, Hefei 230601, China

**Keywords:** low temperature, weak solvation, dilution agent, hybrid electrolytes

## Abstract

Sodium-ion batteries (SIBs) show significant promise for their abundance and potential fast kinetics. However, ether-based electrolytes are limited by low anodic stability, and carbonate electrolytes typically suffer from reduced conductivity and sluggish desolvation at lower temperatures. Here, a weak-solvation dilution strategy that utilizes methyl difluoroacetate (MDFA) as a weakly solvated diluting agent to prepare an optimized carbonate/ester hybrid electrolyte is reported. MDFA reduces the bulk viscosity and takes part in the solvation structures to promote bulk ionic transportation and desolvation. Meanwhile, the reduced interaction between sodium and the solvent system limits salt dissolution to improve the interphase durability. The optimized electrolyte has an energy density of 60 Wh kg^−1^ at −70°C with a full cell using Na_4_Fe_3_(PO_4_)_2_P_2_O_7_ (NFPP) and hard carbon (HC), which exceeds the current limitation of carbonate electrolytes. It also enables Na(Ni_1/3_Fe_1/3_Mn_1/3_)O_2_||HC (NNFMO||HC) pouch cells to maintain 83% of room temperature capacity at −30°C and function with an appliance at −50°C. This strategy provides the possibility of operating high-power SIBs under low temperatures, which could also be extended to other batteries.

## Teaser

A diluting agent features with improved ionic conduction at low temperatures is used to fabricate an ultra-low concentration electrolyte, which supports sodium-ion battery to work at −70°C.

## INTRODUCTION

Sodium-ion batteries (SIBs) have received massive attention for energy storage and start–stop batteries recently. The larger radius of Na^+^ over Li^+^ weakens the binding with solvents and brings a kinetic superiority of SIBs during the rate-determining desolvation process over lithium-ion batteries (LIBs). Therefore, SIBs are ideal for high-rate and low-temperature applications [1–3]. Among components of SIBs, electrolytes are the most vulnerable to low temperatures. Increased viscosity and freezing of electrolytes lead to higher internal resistance, slower ionic transportation, and low capacity and energy at low temperatures. Moreover, sluggish desolvation also halts electrochemical reactions [4,5]. Consequently, there is a high demand for new electrolytes with exceptional adaptability to low temperatures.

Differences in physicochemical properties have become the dominant factor limiting electrolyte selection. An ethylene carbonate (EC) and propylene carbonate (PC) mixture offers better anti-freezing ability than classic cyclic and linear carbonate mixtures [6,7]. Unfortunately, at sub-zero temperatures, viscosity increases as temperature decreases, causes severely weakened ionic transportation [6,8]. A low concentration (0.3 M) strategy was applied to reduce the viscosity of the EC/PC electrolyte to improve performance [9]. On the other hand, strong binding of carbonates with Na^+^ also demonstrates sluggish interphasial kinetics [2]. Opposite to carbonates, most of the ether solvents possess low viscosities, low melting points and better kinetics to work at temperatures below −40°C [10–13]. However, ethers lack the anodic stability to work at high voltage, running counter to the expectation of high energy-density SIBs. Solutions to improve their anodic stability usually come with the sacrifice of low temperature adaptivity [14–17].

Rapid interphasial kinetics and fast ion conduction are also hard to meet all at once. Breakthroughs in solvent engineering have been developed in LIBs. Fluorinated carboxylic esters are reported to show great performance at low temperatures [18,19]. The F-substituted carboxylic esters maintain low viscosities and melting points, which favor ionic transportation at low temperatures. Inspiring, fluorinated carboxylic esters also possess mild dielectric constants and donor numbers to demonstrate weak solvation with lithium ions [20]. These solvents could offer ideal weak cation–solvent interaction with sodium ions, bringing improvement in interphasial kinetics while maintaining good anodic stability.

Herein, we proposed a novel weak-solvation dilution strategy to construct a fluorinated carboxylic ester/carbonate hybrid electrolyte with methyl difluoroacetate (MDFA). The introduction of MDFA resulted in an optimized electrolyte with low melting point (−112°C), ultra-low viscosity (12.3 mPa s at −40°C), wide electrochemical stability window (ESW) (>4.0 V vs. Na/Na^+^) and less solubility of interphase components. The involvement of MDFA in the solvation structure resulted in strong binding components and enhanced cation mobility to exhibit advanced performance at both ambient and extreme temperatures. Specifically, at −70°C, the optimized electrolyte allows the Na_4_Fe_3_(PO_4_)_2_P_2_O_7_||hard carbon (NFPP||HC) full cell to exhibit an impressive specific capacity of 55 mAh g^−1^. The high-voltage Na(Ni_1/3_Fe_1/3_Mn_1/3_)O_2_||HC (NNFMO||HC) pouch cell holds excellent retention and practical significance. Beyond merely reducing the low temperature limitations, this strategy also resolves a challenge that simultaneously mitigates kinetic and ionic transportation for low-temperature applications.

## RESULTS AND DISCUSSION

### Electrolyte design

The strong electron-withdrawing ability of the F atom reduces the electronic density on the coordinated O atom, reducing the solvation power [21]. Comparing fluoroethylene carbonate (FEC) with EC, F-substitution reduces the donor number to dissolve salts but it does not bind to cations too tightly [22,23]. However, weaker solvation comes with increased cluster size and viscosity. For example, the FEC/PC solvent system (brown) presents a weaker solvation ability than EC/PC (red). The mobility of the cation cluster decreases as the cluster size and medium viscosity increase. For pure carbonates, interphasial kinetics and ionic transportation mitigation are always contradictory.

**Scheme 1.**
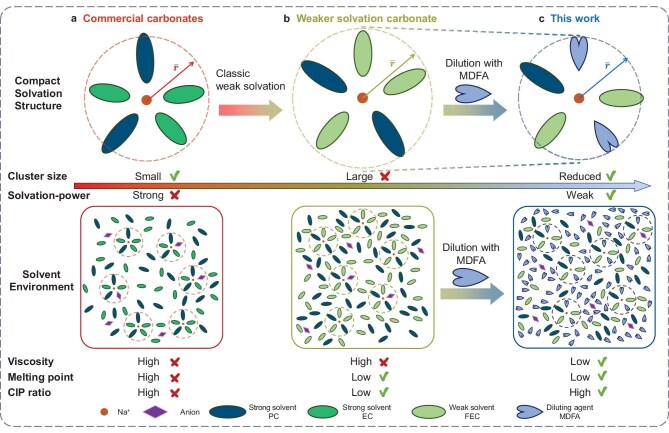
Differences of solvation structures and global electrolyte environments between (a) common carbonate electrolyte, (b) weaker solvation carbonate electrolyte and (c) the electrolyte designed by weak-solvation dilution strategy (this work).

The low melting point (below −90°C) and mild donor number (8.4) are distinctive properties of MDFA for low-temperature batteries [19] but the low dielectric constant (10.1) makes it hard to dissolve sufficient sodium ions for high ionic conductivity (Fig. S1a). Alternatively, utilizing these properties of MDFA as a diluting agent is reasonable for obtaining a weak-solvation electrolyte. Through this weak-solvation dilution strategy, a fluorinated carboxylic ester/carbonate hybrid low-concentration electrolyte, 0.25 M NaClO_4_ in FEC/PC/MDFA (1:1:2 in vol%) (0.25 FPM), is established by using MDFA to dilute 0.5 M NaClO_4_ in FEC/PC (1:1 in vol%) (0.5 FP). 1.0 M NaClO_4_ in EC/PC (1:1 in vol%) (1.0 EP) is used for comparison. In Scheme 1, the low concentration of 0.5 M sodium salt in 0.5 FP reduces viscosity and provides a higher degree of salt dissociation. Therefore, the ratio of strongly binding contact ion pairs (CIPs) decreases. As MDFA is introduced into 0.5 FP, when it replaces the large FEC and PC molecules in the solvation structure (blue), the mobility of the cation cluster increases as the size of the cation cluster reduces. In the solvent pool, MDFA takes 47% of the overall molecules to increase the chance of ‘colliding’ with sodium ions to take part in the solvation. In general, 0.25 FPM is flooded, with a low melting point and free solvent, to effectively separate the cation clusters and anions and improve the mobility of the ionic species. Therefore, an electrolyte with low viscosity, low melting point and weaker solvation power is built to simultaneously enhance the interphasial kinetics and bulk ionic transportation for better performance.

### Electrolyte properties and simulations

**Figure 1.**
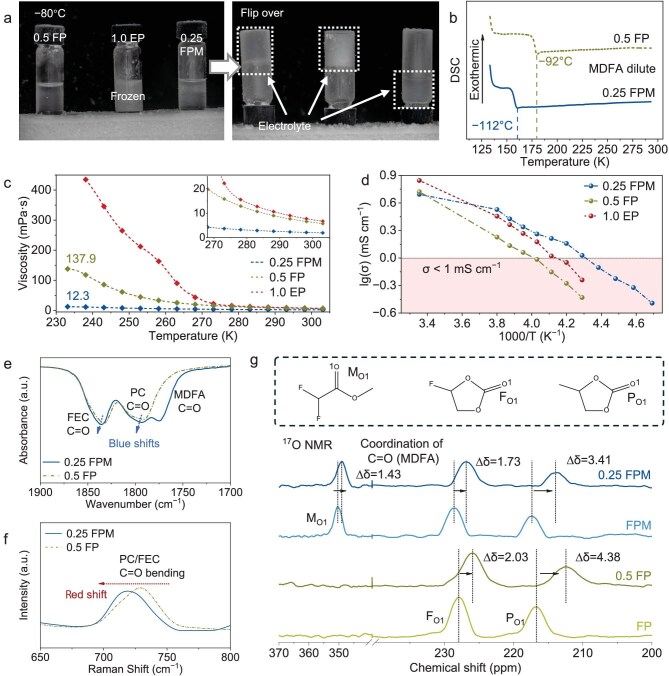
Physicochemical properties of 0.25 FPM and other electrolytes. (a) Optical photographs of 0.5 FP, 1.0 EP and 0.25 FPM at −80°C before and after flip over. (b) DSC curves and glass transition temperature of 0.5 FP and 0.25 FPM. (c) Viscosity curves of 0.5 FP, 1.0 EP and 0.25 FPM from −40°C to 30°C. (d) Ionic conductivity curves of 0.5 FP, 1.0 EP and 0.25 FPM from −60°C to 25°C. (e) FTIR spectroscopy and (f) Raman spectroscopy of 0.25 FPM and 0.5 FP. (g) Molecular structure of MDFA, FEC and PC, and their relative chemical shifts of the carbonyl oxygen in ^17^O NMR spectroscopy of FP, FPM solvent, 0.5 FP and 0.25 FPM.

Solidification and crystallization should never be expected for electrolytes working at low temperatures [24]. In Fig. 1a, 1.0 EP has frozen before the temperature has dropped to −80°C. After flipping over, 0.25 FPM quickly drops to the bottom, and the 0.5 FP presents high viscosity to stay at the top. Differential scanning calorimetry (DSC) was used to confirm the freezing point. The freezing point of 0.5 FP is around −92°C; after dilution with MDFA, that of 0.25 FPM is further pushed to −112°C (Fig. 1b). The MDFA effectively broadens the liquid temperature range of the electrolyte and provides significant viscosity mitigation. As shown in Fig. 1c, 1.0 EP presents the highest viscosity and the sharpest increasing trend. When the temperature reaches −40°C, 0.5 FP displays a high viscosity of 137.9 mPa s, while 0.25 FPM exhibits only 12.3 mPa s. The lowest volumetric concentration of 0.25 FPM leads to the lowest ionic conductivity (4.30 mS cm^−1^) compared with 1.0 EP (7.0 mS cm^−1^) and 0.5 FP (5.28 mS cm^−1^) at 25°C. Surprisingly, the sharp rise in viscosity with increasing salt concentration drives the superiority of 0.25 FPM, such that the ionic conductivity of 0.25 FPM exceeds 1.0 mS cm^−1^, while that of 1.0 EP and 0.5 FP are 0.58 and 0.37 mS cm^−1^ at −40°C (Fig. 1d). Curiously, the low transference number of 0.5 FP experienced a rise after being diluted with MDFA (Fig. S1d) [25]. Within the same solvent set of FPM, 0.15 M and 0.5 M NaClO_4_ (saturated, Fig. S1b) are compared to reveal the effect of concentration on ionic conductivity (Fig. S1c). For 0.15 M, a lack of charge carriers outweighs the improvement of low viscosity, but for 0.5 M, increased viscosity drags the ions more slowly to reduce ionic conductivity. Therefore, 0.25 FPM is currently the prime choice.

Red shifts of the carbonyl bonds (PC and EC: 1800 and 1775 cm^−1^) are observed in Fourier transform infrared (FTIR) spectra (Fig. S2a), while the blue shifts of carbonyl bending vibration (PC and EC: 714 and 717 cm^−1^) and ring symmetric vibrations (PC and EC: 852 and 894 cm^−1^) are observed in Raman spectra (Fig. S2b). These represent the cyclic carbonate molecules being coordinated by sodium ions [26–28]. There is evidence of solvation exhibited in the FTIR and Raman spectra of 0.5 FP and 0.25 FPM (Figs S3 and S4a) [29]. After diluting 0.5 FP with MDFA, the carbonyl peak of FEC and PC in FTIR spectroscopy has shown a blue shift (Fig. 1g), and the carbonyl bending vibration in Raman spectroscopy shows a red shift (Fig. 1f). These shifts suggest partial replacement of FEC and PC with MDFA in the solvation shell. Meanwhile, peaks (921 and 861 cm^−1^) corresponding to the conformer of MDFA solvent present shifting (Fig. S4b) [30]. ^17^O NMR provides direct proof of MDFA solvation (Fig. 1g). After dilution, an upfield displacement of the carbonyl oxygen peak of MDFA appeared, while the upfield displacement of both the carbonyl oxygen of FEC and PC is decreased [31–33]. This evidence proves that MDFA partially substitutes PC or FEC molecules and is involved in the solvation structures to impact the interphasial kinetics.

**Figure 2.**
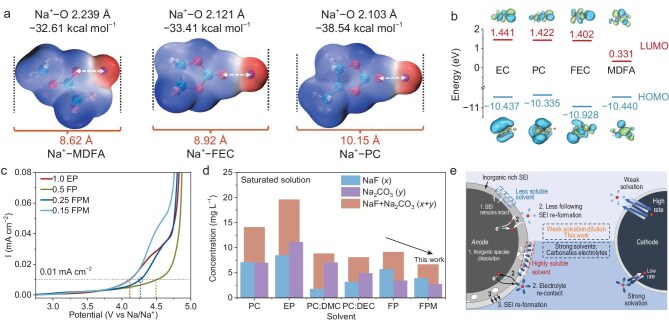
Computational simulation and electrochemical properties of 0.25 FPM and other electrolytes. (a) Binding energies, Na^+^−O bond lengths and electronic cloud lengths of Na^+^−MDFA, Na^+^−FEC, Na^+^−PC’. (b) LUMO and HOMO energy levels of EC, PC, FEC and MDFA. (c) LSV curves of the anodic limit of 0.25 FPM, 0.5 FP and 1.0 EP. (d) NaF and Na_2_CO_3_ dissolution concentrations with PC, EP, PC/DMC, PC/DEC, FP, and FPM solvent. (e) Schematic illustration of interphase stability of weak solvation dilution design.

The weakly solvated nature of the MDFA is confirmed by the density functional theory (DFT) calculation. Figure 2a presents the structures and electronic clouds of the coordinated solvent molecules with sodium ions. When MDFA is coordinated by Na^+^, the Na^+^−O bond length is 2.239 Å and the binding energy of MDFA with Na^+^ is −32.61 kcal mol^−1^. Meanwhile, the F atom of the −CHF_2_ ligand responds to the positive charge and rotates close to the Na^+^, causing the structure of MDFA to turn from the most stable *gauche* to a *cis* conformer and the peak shift of MDFA after solvation (Fig. S4c) [34]. The binding energies of FEC, PC and EC with Na^+^ are −33.41, −38.54 and −37.45 kcal mol^−1^, respectively, and the corresponding Na^+^−O bond lengths are 2.121, 2.103 and 2.107 Å (Fig. S5), indicating stronger cation–solvent interaction. After being coordinated by Na^+^, PC has the largest electronic cloud length (10.15 Å). The electronic cloud and Na^+^−O bond length of FEC are larger (8.92 and 2.121 Å) than those of EC (8.76 and 2.107 Å). MDFA has the smallest electronic cloud length (8.62 Å). When 0.5 FP is diluted by MDFA, the smallest MDFA molecules substitute larger PC and FEC in the solvation structure and decrease the size of the cation clusters, simultaneously forming weaker solvation cation clusters and increasing the mobility, leading to a higher transference number [35].

The MDFA dilution also promotes interphasial kinetics. The ClO_4_^−^ presents much stronger binding with Na^+^ than the solvents, and the solvent-separated ion pair (SSIP) is more kinetically favored than the CIP (Fig. S6). Molecular dynamic (MD) simulation shows the coordination differences between electrolytes [36–38]. The Na^+^−O (ClO_4_^−^) coordination number was reduced in the order of 1.0 EP, 0.5 FP and 0.25 FPM, as the concentration decreased (Fig. S7a). In 0.25 FPM, Na^+^ and ClO_4_^−^ are mostly dissociated (Fig. S8). There is an almost equal ratio of CIP and SSIP in the 1.0 EP; this ratio decreases to 51.3% (0.5 FP) and 19.7% (0.25 FPM) (Fig. S9). Meanwhile, the coordination number of PC and FEC have decreased from 2.26 to 1.47 and from 2.90 to 1.35, respectively. The coordination number of the weakly solvated MDFA is 2.2 (Fig. S7b and S7c). The primary solvation structures are P2F1M2, P1F2M2, P1F1M3 and C1P1F1M2 for 0.25 FPM (Fig. S10). The involvement of MDFA inside clusters improves the interphasial kinetics and brings an F-rich inner side of the clusters, which facilitates NaF formation in the interphases (Fig. S7d).

The stability of MDFA significantly impacts the ESW of 0.25 FPM. According to DFT simulation, MDFA possesses a lower highest occupied molecular orbit (HOMO) energy level compared with PC and EC, which guarantees its anodic stability against high voltage (Fig. 2b) [39]. In the linear sweep voltammetry (LSV) curve, the potentials of 0.5 FP, 0.25 FPM and 1.0 EP are 4.51, 4.27 and 4.12 vs. Na/Na^+^ when the responding current reaches 0.01 mA cm^−2^. The anodic stability of 0.25 FPM is slightly compromised when compared with 0.5 FP (Fig. 2c). The anodic stability is weakened if the salt concentration of the FPM electrolyte is limited to 0.15 M. The cathodic stability of 0.25 FPM also stands in the middle of 0.5 FP and 1.0 EP (Fig. S11).

The stability of electrolytes in cells is affected by interphases, while the stability of the interphases is affected by solvents. A limited salt dissolution rate of the solvent is vital for interphase stability. NaF and Na_2_CO_3_ are two crucial inorganic interphase components to maintain stability. Their dissolution conditions with various solvents are evaluated via inductively coupled plasma optical emission spectrometry (ICP-OES) (Fig. 2d). The dissolution concentration trends demonstrate that strong-binding solvents (e.g. PC and EC/PC) present a higher dissolution concentration than weak-binding solvents (PC/DMC and PC/DEC). The dissolution rate of 0.25 FPM displays a reduced value compared to 0.5 FP. As demonstrated in Fig. 2e, a high dissolution rate of the inorganic component could lead to a loose and electronic conducting interphase, further causing rebuilding and thickening of the solid–electrolyte interphase (SEI). On the contrary, an FPM system has limited dissolution ability to create an interphase-friendly condition, therefore demonstrating its preservation of interphases.

In general, the use of MDFA dilution broadens the liquid range of carbonate electrolytes and reduces the electrolyte viscosity to aid bulk ionic transportation. This hybrid electrolyte provides favorable kinetics for the cathode and a relatively stable interphase environment, which is highly promising for achieving excellent performance.

### Electrolyte performance evaluation

**Figure 3.**
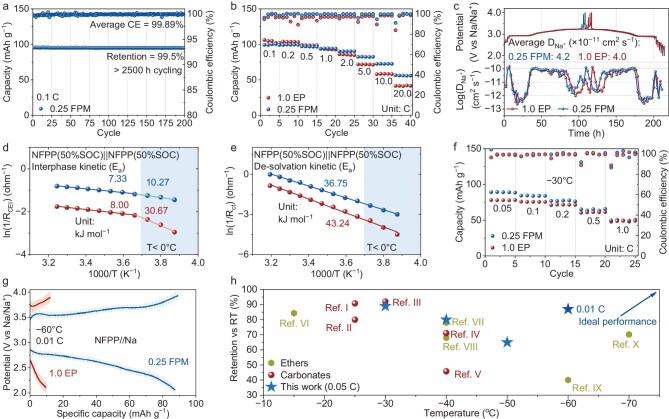
Electrochemical evaluation of 0.25 FPM and 1.0 EP with the NFPP cathode system. (a) Long cycling stability of NFPP||Na at 0.1 C (1 C = 130 mA g^−1^). (b) Rate performance of NFPP with 0.25 FPM from 0.1 to 20.0 C. (c) GITT test of NFPP with 1.0 EP and 0.25 FPM at 0.1 C. (d) Desolvation and (e) interphase kinetics of 0.25 FPM and 1.0 EP derived from EIS of NFPP symmetric cell. (f) Rate performance of NFPP with 0.25 FPM from 0.05 to 1.0 C at −30°C. (g) GCD curve of NFPP at −60°C, 0.01 C with 0.25 FPM and 1.0 EP. (h) Comparison between capacity retention of 0.25 FPM with reported low temperature retention of ethers and carbonates electrolytes. Ref I to V: low temperature retention of ether electrolytes. Ref VI to X: low temperature retention of carbonate electrolytes [7–10,50–55].

Consistent with the LSV results, at 0.1 C (1.0 C = 130 mA g^−1^), an initial Coulombic efficiency (ICE) of 91% is achieved in the initial cycle of NFPP||Na with 0.25 FPM, which is higher than that of 1.0 EP. The low concentration of 0.25 FPM has limited impact on the galvanostatic charge–discharge (GCD) curve, where tiny polarization (8 mV) and discharge capacity (2.0 mAh g^−1^) differences exist (Fig. S12). Meanwhile, 0.25 FPM presents good cycling stability; an average Coulombic efficiency (CE) of 99.89% and capacity retention of 99.50% are achieved after cycling at 0.1 C for 200 cycles (Fig. 3a). In the 1.0 C cycling of NFPP with 0.25 FPM, an average CE of 99.88% and capacity retention of 95.50% is achieved after 500 cycles (Fig. S13). The low viscosity and high kinetics of 0.25 FPM benefit the high-rate performance. NFPP with 0.25 FPM delivers 90.0%, 82.4%, 72.5% and 56.5% of the discharge capacity (0.1 C) at 2.0, 5.0, 10.0 and 20.0 C, which are higher than those of 1.0 EP (Fig. 3b). The GCD curve of NFPP with 0.25 FPM presents slightly higher polarizations at 10.0 and 20.0 C, but the capacity of the plateau region is well preserved by 0.25 FPM, which brings the capacity superiority of NFPP over 1.0 EP (Fig. S14). Cyclic voltammetry (CV) curves of the NFPP were measured at various scan rates, and the ratio of bulk diffusion coefficients were fitted for comparison (Fig. S15). The bulk diffusion coefficient at the anodic peak potential of 0.25 FPM is 2.19-fold that of 1.0 EP. According to the galvanostatic intermittent titration technique (GITT), the average diffusion coefficient of NFPP with 0.25 FPM and 1.0 EP is 4.2 × 10^−11^ and 4.0 × 10^−11^ cm^2^ s^−1^ (Fig. 3c). The higher diffusion coefficient measured benefits from the better wettability of 0.25 FPM (Fig. S16).

Temperature-dependent electrochemical impedance spectroscopy of symmetric cathode cells (Fig. S17a and S17b) was measured to represent the cathode–electrolyte interphase (CEI) diffusion kinetics and desolvation kinetics of 0.25 FPM and 1.0 EP. The diffusion activation energy (E_a_) for both interphases is almost identical at temperatures above 0°C (Fig. 3d). As the temperature falls below 0°C, 0.25 FPM has a lower diffusion E_a_ (10.27 kJ mol^−1^) than 1.0 EP (30.67 kJ mol^−1^). The desolvation E_a_ values of 0.25 FPM and 1.0 EP are 36.75 and 43.24 kJ mol^−1^, respectively (Fig. 3e). Statistically, 0.25 FPM presents a diffusion-friendly CEI and higher desolvation kinetics than 1.0 EP. The Nyquist plots of 0.25 FPM and 1.0 EP half-cells at the 5th to 25th cycles were recorded in Fig. S18a and S18b. The equivalent circuit is displayed in Fig S18c. A higher slope of NFPP with 0.25 FPM in the low-frequency region demonstrates better diffusion ability (Fig. S18d). The contrast between impedance values indicates an evolutionarily stable CEI and higher overall kinetics of 0.25 FPM than 1.0 EP (Fig. S18e).

0.25 FPM is robust enough to endure a high potential of 4 V vs. Na/Na^+^ and works well with the layered NNFMO. After cycling at 1.0 C for 200 cycles, the NNFMO with 0.25 FPM exhibits an average CE of 99.87% and capacity retention of 94.56%, which are all higher than that of 1.0 EP (Fig. S19). NNFMO with 0.25 FPM delivers higher capacity at 5.0 and 10.0 C, which also confirms the kinetics superiority of 0.25 FPM (Fig. S20).

0.25 FPM presents advanced performance at low temperatures. For the NFPP cathode, 0.25 FPM delivers 89.4% (0.05 C), 84.0% (0.1 C) and 77.5% (0.2 C) of its discharge specific capacity at 0.1 C and 25°C. In the same conditions, 1.0 EP only delivers 74.2%, 72.0% and 68.3%, respectively (Fig. 3f). The lengths of the plateau capacity at multiple rates are well preserved by 0.25 FPM (Fig. S21). 0.25 FPM provides good stability at −30°C. At 0.1 C, NFPP with 0.25 FPM shows an unfaded capacity and an average CE of 99.49% (Fig. S22a). Stability of 0.25 FPM remains at a higher rate. After cycling at 0.5 C for 250 cycles, the capacity retention of NFPP with 0.25 FPM is 95.1% with an average CE of 99.89% (Fig. S22b). The superiority of 0.25 FPM remains for both NFPP and NNFMO at −30°C. Figure S23a displays the cycling stability of the NNFMO with 0.25 FPM and 1.0 EP at −30°C and 0.1 C. After 70 cycles, 0.25 FPM preserves a capacity retention of 85.7% but that of 1.0 EP preserves only 45.8%. The length of the plateau capacities of the NNFMO with 0.25 FPM is also well preserved (Fig. S23b and S23c). Differences in rate performance between NNFMO with 0.25 FPM and 1.0 EP at −30°C are more obvious and the general rate capability of the NFPP is better than the NNFMO at low temperatures (Fig. S24).

For pure carbonate electrolytes, the current low-temperature limit is at around −40°C. The dilution by MDFA makes a breakthrough. 0.25 FPM is capable of working even at a harsher condition of −60°C. In Fig. 3g, a discharge specific capacity of 87 mAh g^−1^ is delivered at 0.01 C, while the frozen 1.0 EP only delivers 10 mAh g^−1^ with larger polarization. GCD curves of 0.25 FPM at 0.05 C@−40°C, 0.05 C@−50°C and 0.02 C@−60°C are displayed in Fig. S25a. An intact GCD curve is exhibited at −40°C, while the high polarization is the capacity-limiting factor at −50°C and −60°C. This fluorinated carboxylic ester/carbonate hybrid electrolyte can work at much lower temperatures than pure carbonate electrolytes and narrow the performance gap with ether electrolytes (Fig. 3h). From the stability perspective, the anti-oxidation superiority of 0.25 FPM provides superior compatibility of high-voltage cathodes over the others, demonstrating a combination of low-temperature performance and stability (Fig. S26).

### Interphase properties

**Figure 4.**
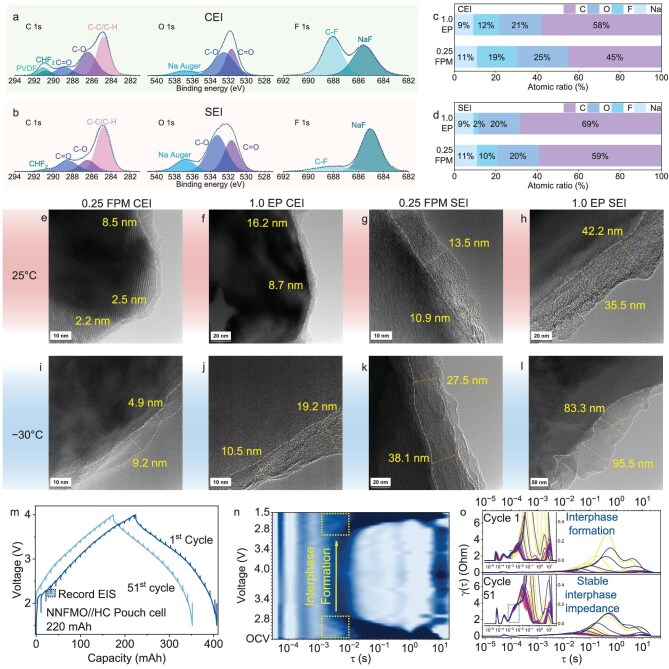
Interphase condition of 0.25 FPM and 1.0 EP in NFPP||HC. C 1s, O 1s and F 1s XPS spectra of (a) CEI and (b) SEI in NFPP||HC with 0.25 FPM after 100 cycles. Atomic ratio statistics of (c) CEI and (d) SEI in NFPP||HC with 0.25 FPM and 1.0 EP after 100 cycles. HRTEM of CEI of (e) 0.25 FPM and (f) 1.0 EP, and SEI of (g) 0.25 FP and (h) 1.0 EP generated at 25°C. HRTEM of CEI of (i) 0.25 FPM and (j) 1.0 EP, and SEI of (k) 0.25 FPM and (l) 1.0 EP generated at −30°C. (m) GCD curve and *in*  *situ* EIS measuring points of NNFMO||HC pouch cell with 0.25 FPM (1st and 51st cycle). (n) DRT-state of charge (SOC) contour mapping derived by *in*  *situ* EIS at the first cycle. (o) DRT comparison between NNFMO||HC pouch cell with 0.25 FPM at 1st cycle and 51st cycle; inset enlarges the γ(τ) axis.

Interphase-forming ability is critical for electrolytes to work in full cells and the quality of the interphases is fundamental to their cycling life. Figure 4a and b shows the X-ray photoelectron spectroscopy (XPS) spectra of CEI and SEI after 100 cycles. NaClO_4_ is relatively stable and merely affects the component of the interphase; the CEI and SEI are mostly solvent-dominated (Fig. S27). In the C 1s spectra, the CEI of 0.25 FPM and 1.0 EP (Fig. S28a) has a polyvinylidene difluoride (PVDF) peak, which comes from the binder of the cathode. A small peak around 291 eV only shows in the spectra with 0.25 FPM, which corresponds to the −CHF_2_ peak. It comes from the decomposition of MDFA at both the cathode and anode surface [20]. Peaks at 289, 286.5 and 284.5 eV correspond to the C=O, C−O and C−C/H bonds [40]. The C−C/H bond intensities of 0.25 FPM at both the CEI and SEI are lower than that of 1.0 EP (Fig. S28a and S28b) and this is also reflected in the atomic ratio count in Fig. 4c and d. A higher Na ratio and lower C ratio are observed on the CEI and SEI of 0.25 FPM. The CEI and SEI generated by 0.25 FPM both have fewer organic components. Another difference is the F component, where F accounts for 12% of the atom count in 1.0 EP CEI, but the only F source in the CEI of 1.0 EP is the PVDF (66%) and PVDF decomposition product (34%) (Fig. S29) [41]. In the CEI of 0.25 FPM, F accounts for 19% of the atom count, and 52% of the F atoms come from the NaF (Fig. 4a). Owing to the close contact of Na and F in the solvation structures and the easily reduced nature of MDFA, NaF could easily be generated and help to build robust interphases [42].

The morphology of the interphases is observed by high-resolution transmission electron microscopy (HRTEM). The F-rich CEI of 0.25 FPM (2–8 nm) is thinner than that of the 1.0 EP (8–16 nm) (Fig. 4e and f). As the side reactions happen mostly at the anode side, the difference in the thickness of SEI is more obvious. The thickness of the 0.25 FPM SEI is 10–15 nm, while that of the 1.0 EP SEI is 35–45 nm (Fig. 4g and h). Both the CEI and SEI generated by 0.25 FPM are robust at preventing further side reactions against high voltage, and thin to reduce the diffusional barricades. 0.25 FPM also provides a solvent environment that is favorable for preventing interphase dissolving and growing. Cells were further put into −30°C for observing the interphase condition. Owing to high polarization and disfavored thermodynamics, the SEI and CEI formed directly at −30°C are thicker than those at 25°C. The thickness of the CEI of 0.25 FPM is 5–9 nm, thinner than that of 1.0 EP (10 –20 nm) (Fig. 4i and j). The thicknesses of the SEI formed at −30°C of 0.25 FPM and 1.0 EP are 17–40 and 30–96 nm, respectively. In general, interphases of 0.25 FPM are relatively more favorable for diffusion, which is also in accordance with previous impedance results.

*In*  *situ* electrochemical impedance spectroscopy (EIS) was also measured at the 1st and 51st cycles of the NNFMO||HC pouch cell (Fig. 4m). The Nyquist plots within these two cycles show small and overlapped semi-circles. However, the interphase and charge-transfer impedances are hard to precisely distinguish via traditional fitting (Fig. S30a). The distribution of relaxation time (DRT) technique was used to fully decouple the interphase and charge transfer process [43,44]. In Fig. 4n, the contour map displays the DRT of the initial cycle at different voltage stages, where a reversible pattern has been shown from charge to discharge. The irreversible change of DRT appears within the initial cycle at the time constant between 10^−3^ and 10^−2^ s, which is assigned as the region for interphase impedance. Increased color depth revealed the increased interphase impedance after charging. Figure 4o presents the DRT result of the 1st and 51st cycles; the visible differences exhibited between the start and end curves indicate the formation of the interphases. The DRT plot of the 51st cycle presents a higher but unchanged impedance in the interphase region. Interphases within and after the 1st cycle are still forming but fully developed ahead of the 51st cycle. This result is also consistent in the high-frequency region in the Nyquist plot (Fig. S30b). Such stability and the low interphase dissolution rate of 0.25 FPM (Fig. 2d) are highly correlated. The good reversibility demonstrated in the Nyquist plots and DRT plots indicates that the interphase formed by 0.25 FPM exhibited good quality and stability.

### Full cell and pouch cell performances

**Figure 5.**
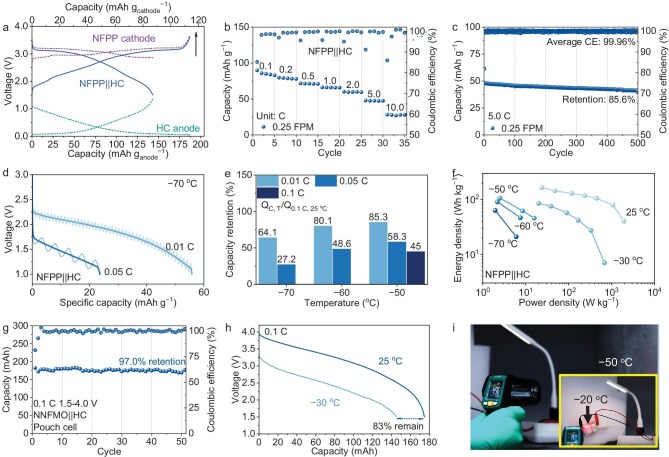
Prototype NFPP||HC full-cell and NNFMO||HC pouch cell performance with 0.25 FPM. (a) GCD curve of NFPP||HC with 0.25 FPM at 0.1 C measured via Swagelok tri-electrode cell. (b) Rate performance and (c) 5.0 C long cycling test of NFPP||HC with 0.25 FPM. (d) Discharge capacities and curves of NFPP||HC at −70°C. (e) The ratio of NFPP||HC discharge capacity at 0.01, 0.05 and 0.1 C (−50 to −70°C) to that at 0.1 C (25°C). (f) Ragone plots of NFPP||HC at various rates and temperatures. (g) Cycling performance of commercial 220 mAh NNFMO||HC pouch cell with 0.25 FPM at 0.1 C. (h) Comparison of discharge curve between NNFMO||HC pouch cell at −30°C and 25°C, 0.1 C. (i) Optical photograph of two series-linked NNFMO||HC pouch cells with 0.25 FPM lighting up 5.0 V commercial LED light at −50°C.

With 0.25 FPM, HC delivers 300 mAh g^−1^ at 10 mA g^−1^, with an ICE of 72%. The discharge capacity of the HC||Na half-cell decreased significantly at higher current densities due to the disappeared plateau, accompanied by large overpotentials (Fig. S31a and S31b). For avoiding Na plating and high-rate purpose, the prototype full cell is thus designed to have excess HC. NFPP is used as the cathode to provide sufficient working ability at low temperatures, and this prototype full cell is denoted as NFPP||HC; the prototype cell has an initial discharge capacity of 87vmAh g^−1^ at 0.1 C (Fig. 5a).

NFPP||HC delivers 80, 72, 66, 60, 48 and 28 mAh g^−1^ at 0.2, 0.5, 1.0, 2.0, 5.0 and 10.0 C (Fig. 5b). There is no obvious polarization on the discharge curve from 0.1 to 2.0 C, which indicates that 0.25 FPM provides good kinetic performance at various rates (Fig. S32). After cycling 200 cycles at 1.0 C, the capacity retention of NFPP||HC is 81% and the average CE is 99.85% (Fig. S33). 0.25 FPM also has an extraordinary fast-charging ability at NFPP||HC. After 500 cycles at 5.0 C, a high retention of 85.5% accompanied by an ultra-high average CE of 99.96% is achieved (Fig. 5c). This rate at NFPP||HC is equivalent to a power density of 1080 W kg^−1^ and an energy density of 80 Wh kg^−1^. In addition, 0.25 FPM also supports the operation of a high area loading (1 mAh cm^−2^) NFPP||HC full cell (Fig. S34).

0.25 FPM enables NFPP||HC to work at low temperatures. At −30°C, 55, 50, 40, 32 and 22 mAh g^−1^ are delivered at 0.1, 0.2, 0.5, 1.0 and 2.0 C (Fig. S35). Compared with room temperature, 65% discharge capacity is available at 0.1 C. Sufficient rate performance is realized by 0.25 FPM at room temperature. Charging at low temperatures still poses a threat to batteries. NFPP||HC is charged at 0.05 C under −30°C and discharged at 0.5 C; the discharge capacity fades from 38 to 23 mAh g^−1^, but with an average CE equal to 100% (Fig. S36). The contradiction between high CE but low capacity-retention is caused by detrimental sodium plating at the HC side. The high polarization occurs to make Na ions start to plate while intercalating, resulting in side reactions and continuous Na ion consumption. Therefore, charging at low temperatures is not encouraged. To fully reveal the potential of the 0.25 FPM at lower temperatures, discharge tests of NFPP||HC are performed after charging at 25°C. At −70°C, and 0.01 and 0.05 C, NFPP||HC displays a discharge specific capacity of 55 and 24 mAh g^−1^, equivalent to a retention of 64.1% and 27.2% of the discharge specific capacity at 25°C and 0.1 C (Fig. 5d). The difference between the GCD curves is the initial discharge voltage (Fig. S37a and S37b). At −60°C, and 0.01 and 0.05 C, NFPP||HC shows a discharge capacity retention of 80.1% and 48.6%, while at −50°C, and 0.01, 0.05 and 0.1 C, the retention of NFPP||HC is 85.3%, 58.3% and 45% (Fig. 5e). In general, the prototype NFPP||HC presents fast charging ability at 25°C and working ability at extreme temperatures. At 25°C, a maximum energy density of 165 Wh kg^−1^ and power density of 2000 W kg^−1^ can be achieved. At −30°C, the maximum power density could reach 680 W kg^−1^. Even at an extreme temperature of −70°C, this battery still possesses an energy density of 60 Wh kg^−1^ (Fig. 5f).

0.25 FPM was filled into pouch cells (220 mAh NNFMO||HC) for further testing. 0.25 FPM demonstrates the best wettability between electrolyte and separator among the group (Fig. S38). Initial discharge capacity of the pouch cell with 0.25 FPM is 180 mAh at 0.1 C in the voltage range of 1.5–4 V, which equals 100.7 Wh kg^−1^. After 50 cycles, a capacity retention of 97% is obtained (Fig. 5g). Furthermore, at 0.5 C, this pouch cell delivers an initial discharge capacity of 150 mAh and it also enables a capacity retention of 70.7% (average CE 99.71%) after 150 cycles (Fig. S39a). The low-temperature working ability of 0.25 FPM is also effective for the pouch cell. The NNFMO||HC pouch cell was taken to −30°C for the discharge test. It holds an available discharge capacity of 145 mAh (83% of that at 25°C) and 63.2 Wh kg^−1^ at 0.1 C (Fig. 5h, Table S1). Compared with publications regarding pouch cells discharging at low temperatures, 0.25 FPM enables top-tier retention (Table S2) [45–49]. To further reveal the potential of 0.25 FPM, the pouch cell was tested with the appliance at extreme conditions. Two series-linked pouch cells light up a commercial 5.0 V LED even at an environmental temperature of −50°C. As the thermal effect of discharge current heating up the pouch cell to −20°C, the LED can output a higher power (Fig. 5i).

## CONCLUSION

In this work, the weak-solvation dilution strategy is successfully employed to establish a low-concentration fluorinated carboxylic ester/carbonate hybrid electrolyte, 0.25 FPM, which features a low melting point, low viscosity, wide ESW, low interphase dissolution and exceptional performance at low temperatures. This strategy simultaneously and jointly promotes bulk ionic transportation, desolvation and interphasial kinetics of carbonate-based electrolytes, enabling cells to function at low temperatures. The F-rich, thin but robust interphases generated by 0.25 FPM are protective and diffusion-friendly. The reduced inorganic salt dissolution rate of 0.25 FPM also guarantees the stabilization of interphases. Meanwhile, the low-temperature capacity retention of 0.25 FPM stands in the top tier among the reported electrolytes. At 25°C, prototype NFPP||HC with 0.25 FPM achieves a maximum power density of 2000 W kg^−1^ (40 Wh kg^−1^). Even at an extremely low temperature of −70°C, this SIB outputs an energy density of 60 Wh kg^−1^. With 0.25 FPM, the NNFMO||HC pouch cell maintains a high capacity retention of 83% and energy retention of 65% at −30°C. This strategy offers new insights into tuning ionic transportation and interphasial kinetics of electrolytes for extreme temperature applications.

## Supplementary Material

nwaf510_Supplemental_File
