# Supplementary material for: Weak-solvation dilution for interphase preservation and extreme low-temperature sodium-ion storage
Source: Natl Sci Rev. 2025 Nov 17;13(1):nwaf510. doi: 10.1093/nsr/nwaf510 (PMC12796803; doi:10.1093/nsr/nwaf510)
Supplement: nwaf510_Supplemental_File [file nwaf510_supplemental_file.pdf]

Supporting Information

Weak-solvation dilution for interphase preservation and extreme low-temperature sodium-ion storage

Jinyu Yang[1,2,‡], Mingxu Wang[1,‡], Haoran Ji[1], Ziyue Li[1], Fengmei Wang[1], Zihao Zhang[1], Xinjie Li[1], Yanru Yang[1], Qin Li[1], Jiafeng Ruan[1,4], Fang Fang[1,2,3], Dalin Sun[1], Fei Wang[1,3,*]*

[1]*College of Smart Materials and Future Energy, Fudan University, Shanghai, 200433, China*

[2]*Yiwu Research Institute of Fudan University, Yiwu City, 322000, China*

[3]*School of Materials Science and Engineering, Anhui University, Hefei, 230601, China*

[4]*Inorganic Chemistry I, Technische Universität Dresden, Dresden 01069, Germany*

[‡]*These authors contributed equally: Jinyu Yang, Mingxu Wang*

Corresponding author:

rjfeng@fudan.edu.cn

feiw@fudan.edu.cn

Experimental

Material and electrolyte Characterization

Dissolution concentration of the salts were measured by Inductively Coupled Plasma-Optical Emission Spectrometer (ICP-OES, Agilent 720ES(OES)). The surface component of cathode and anode was analyzed by X-ray photoelectron spectroscopy (XPS, Thermo Scientific K-Alpha+) with mono Al Kα radiation at 100 eV. The morphologies and structures of the NFPP cathode and HC anode were investigated by High-resolution transmission electron microscope (HRTEM, FEI TALOS F200X). The sodium-solvent interactions were analyzed by Fourier-transform infrared spectroscopy (FTIR, Thermo Scientific Nicolet iS20), Raman spectroscopy (Horiba HR Evolution) using an excitation wavelength of 633 nm and NMR (Bruker AVANCE NEO 600M). Electrolyte viscosities were measured by Thermo HAAKE MARS 60. Contact angles were measured by Dataphysics OCA 25.

Electrochemical measurement

The negative electrode hard carbon (Kuraray) and positive electrode material $Na_4Fe_3(PO_4)_2(P_2O_7)$ (NFPP) and $Na(Ni_{1/3}Fe_{1/3}Mn_{1/3})O_2$ (NNFMO) was purchased. The anode slurry was prepared by blending hard carbon, acetylene black and sodium polyacrylate (Na-PAA, 45% solution, Sigma-Aldrich) at weight ratio of 8:1:1 in water. The cathode slurry was prepared by blending active material (NFPP and NNFMO), Acetylene black and polyvinylidene fluoride (PVDF, 99%, DoDochem) at weight ratio of 8:1:1 in N-Methylpyrrolidone (NMP, 99%, Sigma-Aldrich). Cathode and anode mixtures were dispersed with KURABO MAZERUSTAR KK-250SE Planetary Mixer and pasted onto aluminum foil. The prepared electrodes were dried at 120 °C for 12 hours in a vacuum drying oven. Both cathode and anode were then cut into disk of 12 mm diameter. The 1.0 EP electrolyte were prepared by dissolving 1.0 M $NaClO_4$ (99%, Sigma-Aldrich) into ethylene carbonate (EC, 99%, Sigma-Aldrich):propylene carbonate (PC, 99%, Sigma-Aldrich). 0.5 FP electrolyte were prepared by dissolving 0.5 M $NaClO_4$ into fluoroethylene carbonate (FEC, 99%, DoDochem):PC (1:1 in volume). 0.25 FPM were prepared by mixing 0.5 FP with methyl difluoroacetate (MDFA, 98%, Meryer) at volume ratio of 1:1. All the solvents were pre-treated with molecular sieves for at least 7 days to remove water. Half-cell were assembled by coupling NFPP (5 mg cm^{-2}) /NNFMO (5 mg cm^{-2}) /HC (3 mg cm^{-2}) with sodium disks with GF/A membrane and 100 μL electrolyte. NFPP//HC full-cell were assembled by NFPP cathode (8 mg cm^{-2}) and pre-cycled HC anode (5 mg cm^{-2}) with GF/A membrane and 100 μL electrolyte. The HC is pre-cycled via half-cell, 10 mA g^{-1} for 3 cycles. Energy density is evaluated by the overall mass of the cathode and anode. Dry 220 mAh NNFMO//HC pouch-cell were purchased from Li-FUN and injected with 1 g electrolyte. All cells were assembled and disassembled in argon-filled glove

box, with water and oxygen contents lower than 0.01 ppm. The galvanostatic charge/discharge tests were performed on the battery test system (LANHE CT3002A). The linear sweep voltammetry (LSV), cyclic voltammetry (CV) and Electrochemical Impedance Spectroscopy (EIS) were recorded by BioLogic VSP Potentiostat electrochemical workstation. The energy density of coin cell is evaluated via dividing the tested energy with the overall mass of cathode and anode of the coin cell [Energy/(m_{Ca}+m_{An})]. The energy density of pouch cell is evaluated via dividing the tested energy with full cell mass (Energy/m_{Cell}).

Computational Methods

Quantum chemistry calculations were first performed to optimize molecular geometries of EC, FEC, PC and MDFA solvent molecules, and ClO_4^- anion using the Gaussian 16 package at B3LYP/6-311+G(d,p) level of theory. The atomic partial charges on these solvent and ion molecules were calculated using the ChelpG method at the same level of theory (the B3LYP hybrid functional and and the 6-311+G(d,p) basis set) The atomistic force field parameters for all ions and molecules are described by the AMBER format.[1] The force field parameters for ClO_4^- anion were taken from the previous work and were further calibrated followed the right procedures so as to reproduce physical parameters determined from experimental observations.[2] The cross-interaction parameters between different atom types are obtained from the Lorentz-Berthelot combination rule. All atomistic simulations were performed using GROMACS package with cubic periodic boundary conditions.[3] Binding energy and molecule structures were calculated by SMD solvent model and optimized with M06-2X functional and def2TZVP basis set via Orca. DFT-D3 dispersion correction was used to describe the dispersion interaction.

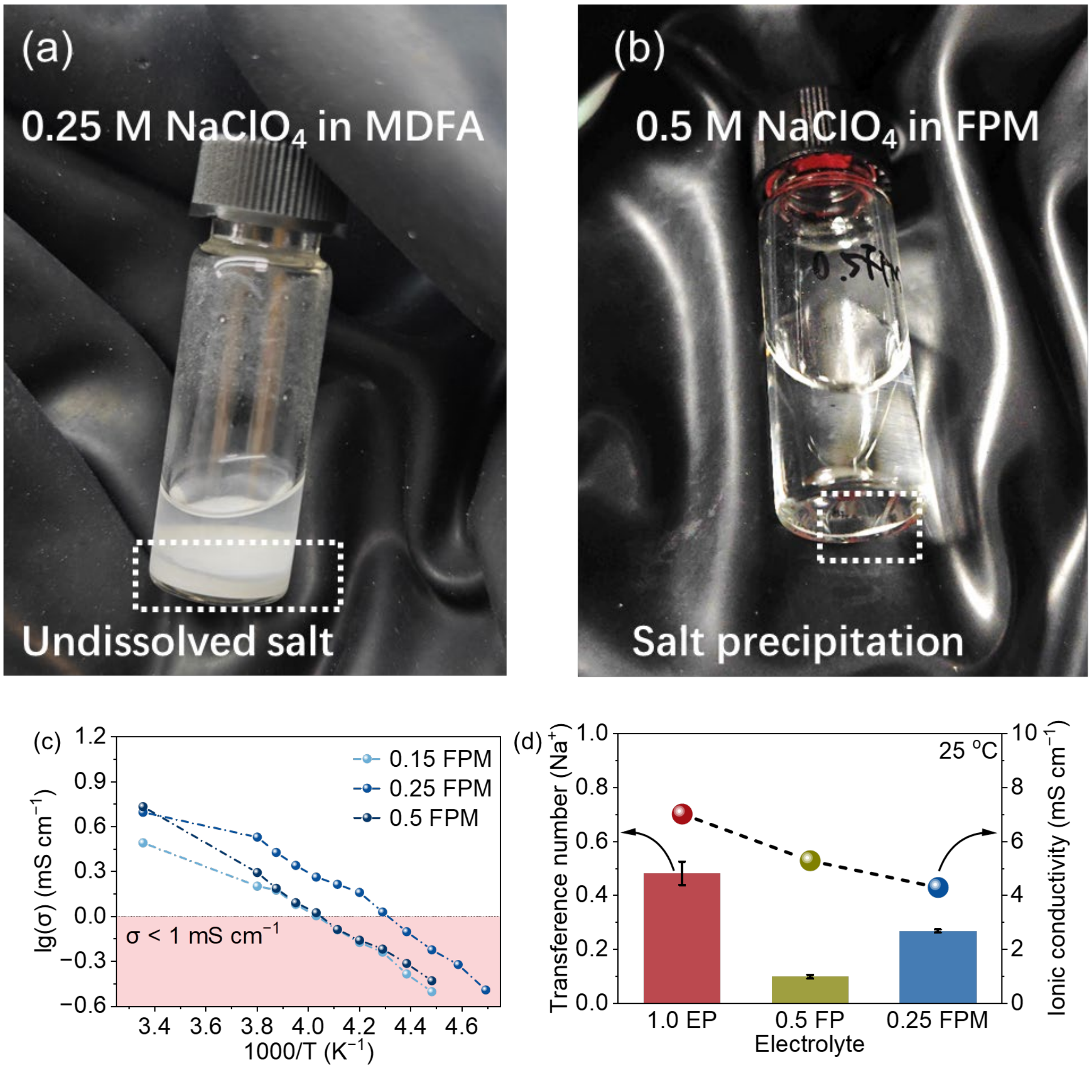

Figure S1. Optical photo of (a) 0.25 mmol $NaClO_4$ with 1 mL MDFA solvent, (b) 0.5 M $NaClO_4$ in FPM, (c) Ionic conductivities curves of 0.5 FPM (Sat.), 0.25 FPM, and 0.15 FPM electrolyte from −60 ºC to 25 ºC, (d) Transference number obtained by Bruce-Vincent method.

Due to small dielectric constant and DN number, strong salt like $NaClO_4$ is hard to dissolve into pure MDFA solvent. 0.5 M $NaClO_4$ in FPM is obtained by diluting 1.0 M $NaClO_4$ in FEC:PC (1:1 in Vol%) with MDFA, after dilution, the electrolyte presents cloudy and salt precipitates after it set for still.

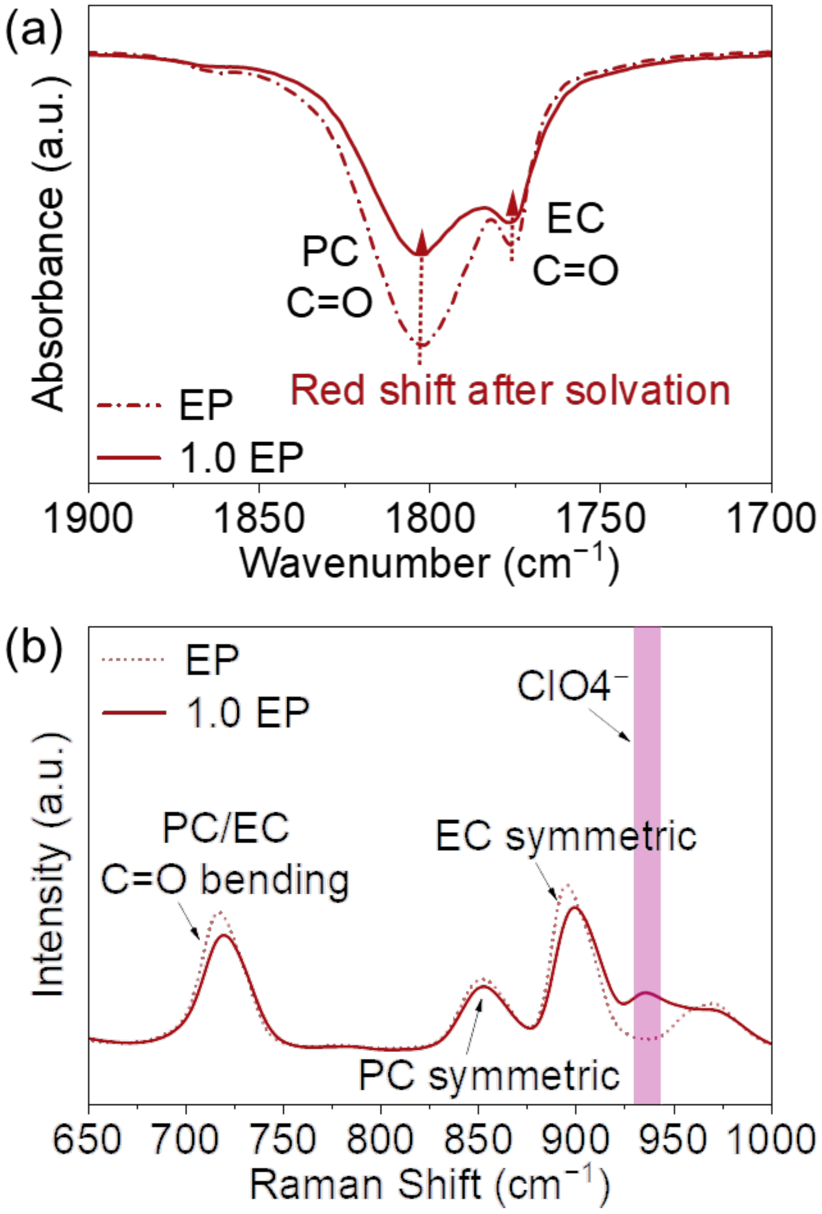

Figure S2. (a) FTIR spectroscopy and (b) Raman spectroscopy of 1.0 EP electrolyte.

In both FTIR and Raman spectroscopy, the shift of wavenumber and Raman shift after solvation are tiny. In FTIR, red shift of carbonyl bond indicates solvation behaviour. In Raman spectroscopy, the pink bar at around 930-940 cm^{-1} indicates the existence of free ClO_4^- ions. The blueshift happened on the EC and PC carbonyl bond bending and ring symmetric vibration is the typical proof of cation solvation.

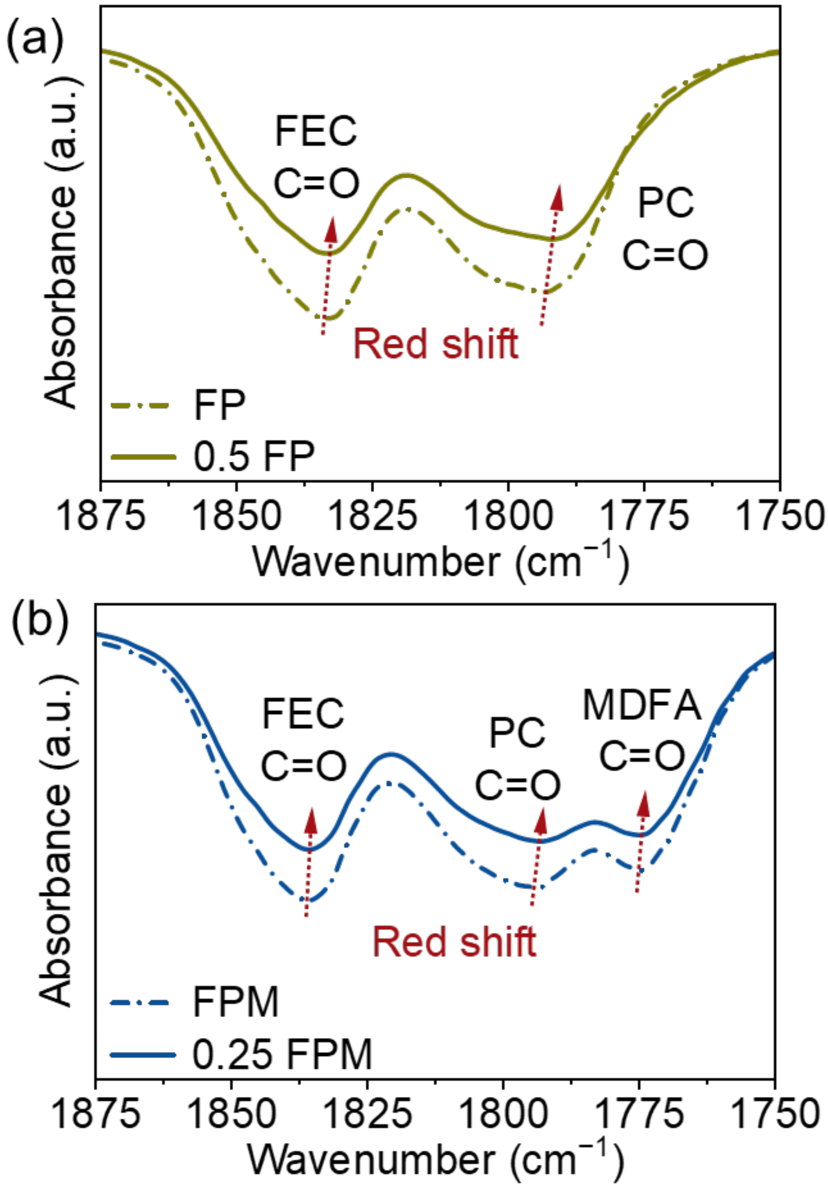

Figure S3. FTIR spectroscopy of (a) FEC:PC (FP) solvent, 0.5 FP electrolyte and (b) FEC:PC:MDFA (FPM) solvent, 0.25 FPM electrolyte.

In the FTIR results, the red shift trend of carbonyl bonds in 0.5 FP and 0.25 FPM are identical to the 1.0 EP.

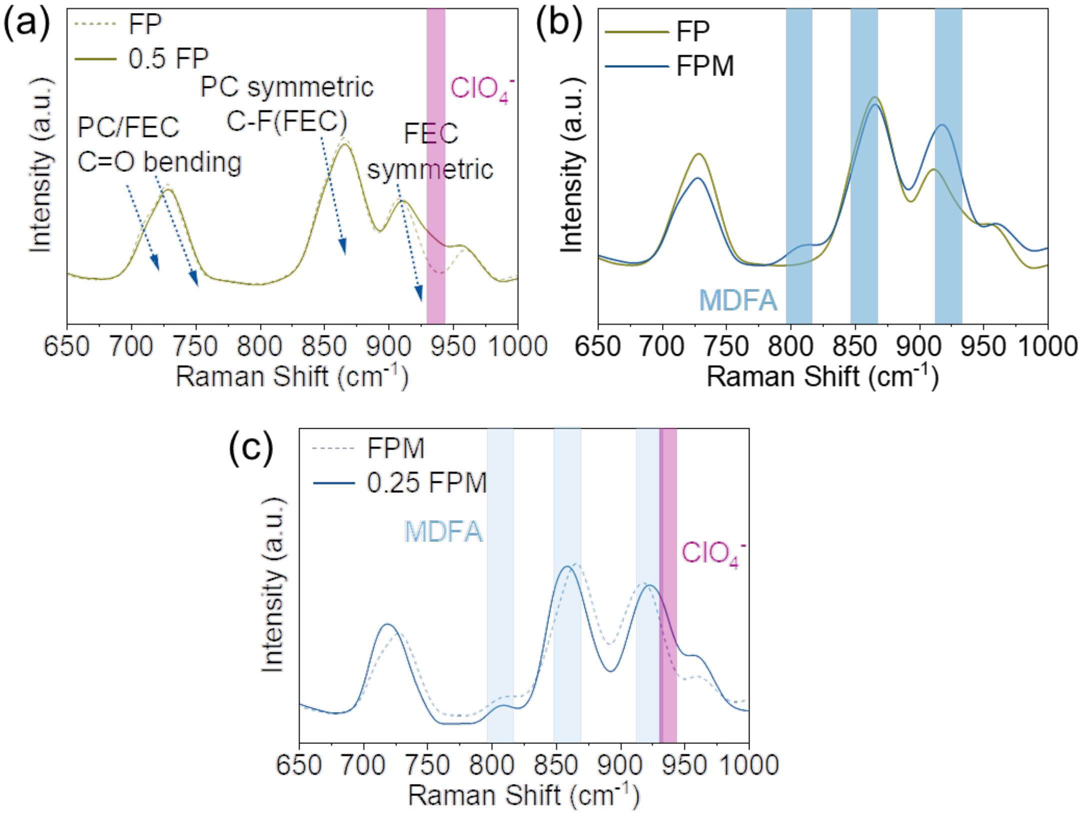

Figure S4. Raman spectroscopy of (a) FEC:PC (FP) solvent, 0.5 FP electrolyte and (b) FEC:PC (FP) solvent, FEC:PC:MDFA (FPM) solvent. (c) FPM solvent and 0.25 FPM

In Raman spectroscopy of 0.5 FP, blueshift of certain bonds are observed. Strong peak shows around 865 cm^{-1} is the coupling of the PC ring symmetric vibration and C-F bond vibration of FEC. In the comparison of FP and FPM, there are 3 typical peaks related to MDFA have marked in blue bands. This bands are conformer related in previous reports. The *gauche* form is the most stable one.

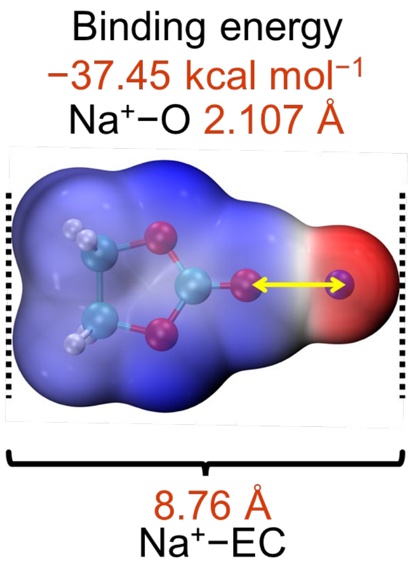

Figure S5. Binding energy, Na^+−O length and largest electronic cloud length of Na^+−EC.

According to Stokes−Einstein equation:

$$\mu_+ = \frac{z_+ e_0}{6\pi\eta} \frac{1}{r_+} \tag{1}$$

$$\mu_- = \frac{z_+ e_0}{6\pi\eta} \frac{1}{r_-} \tag{2}$$

When the cation has a larger solvation sheath, the transport number of cation will decrease

$$t_+ = \frac{\mu_+}{\mu_+ + \mu_-} \tag{3}$$

Therefore, a smaller solvation structure is beneficial to improve cation transport number.

Considering MDFA replace PC and FEC in the 0.25 FPM, the average radius of the solvation structures is smaller than the 0.5 FP to increase the transport number.

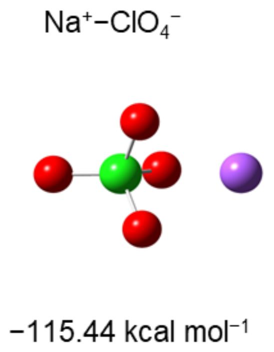

Figure S6. Binding energy of sodium ion with ClO_4^- anion

The strong interactions between ClO_4^- and sodium ions indicate that SSIP are more kinetic favoured than CIP in this case, therefore the reduction of concentration is helpful.

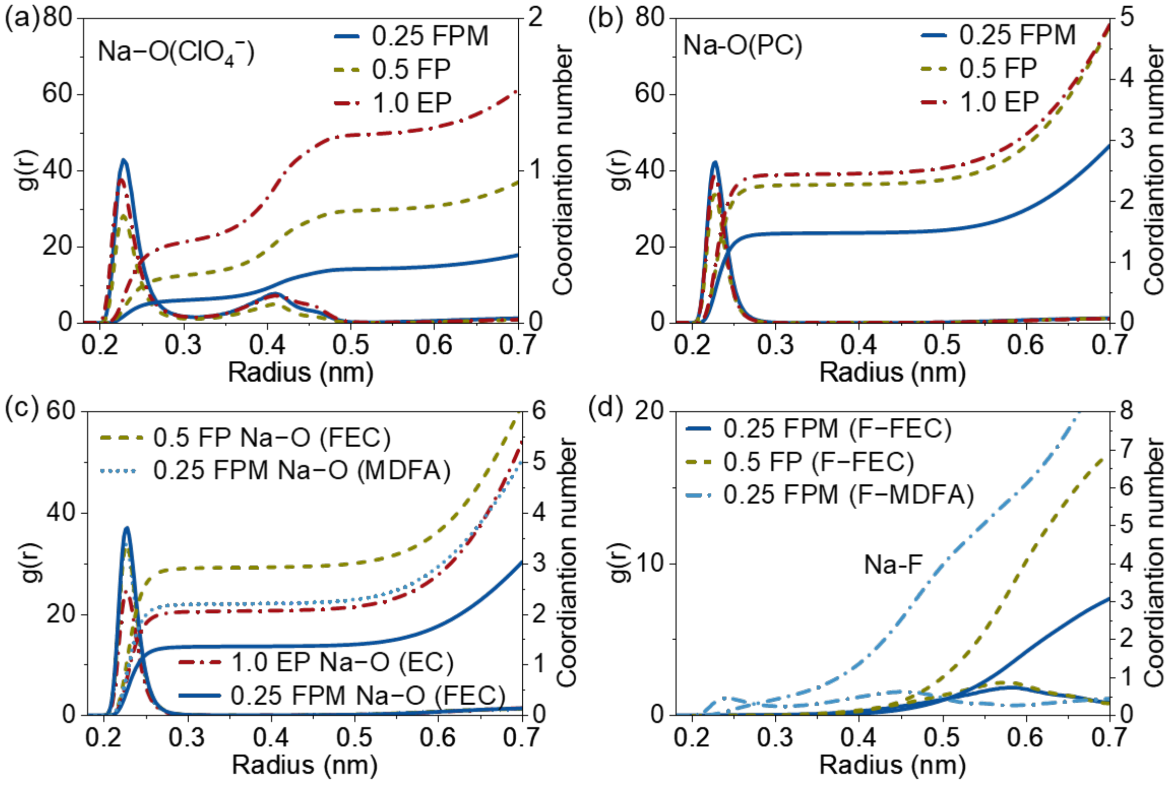

Figure S7. Radius distribution function and coordination number of (c) Na−O ($ClO4^-$) of 0.25 FPM, 0.5 FP and 1.0 EP, (d) Na−O (PC) of 0.25 FPM, 0.5 FP and 1.0 EP, (e) Na−O (FEC) of 0.25 FPM and 0.5 FP, Na−O (MDFA) of 0.25 FPM, Na−O (EC) for 1.0 EP, (f) Na−F (FEC) of 0.25 FPM and 0.5 FP, Na−F (MDFA) of 0.25 FPM.

From the Radius distribution function result, 1.0 EP has a shorter bond length of Na−O (ClO_4^-), Na−O (PC) and highest CN of ClO_4^- among the three electrolytes. The MDFA solvent has been solvated and brings an F-rich environment to the solvation structure. The existence of F atom starts from about 0.25 nm also reflected the change of form for the MDFA. F atom has been attracted by sodium ions, where the *gauche* can turn into *cis* upon solvation.

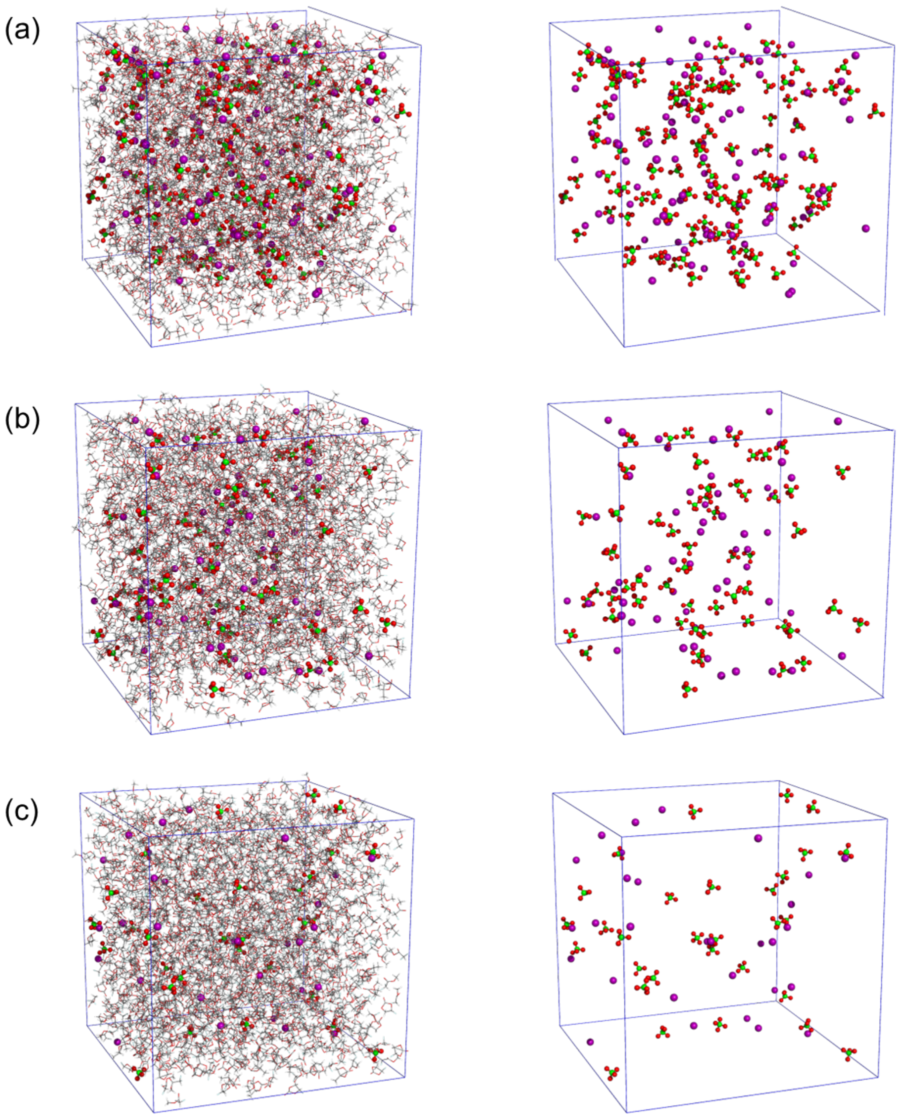

Figure S8. Snapshot of (a) 1.0 EP, (b) 0.5 FP and (c) 0.25 FPM electrolyte with and without solvent molecules. Sodium ions and ClO_4^- anions in snapshot of 1.0 EP are crowded distributed, there are high ratio of CIP. The CIP ratio has decreased in 0.5 FP. For 0.25 FPM, the sodium ions and ClO_4^- anions are fully separated.

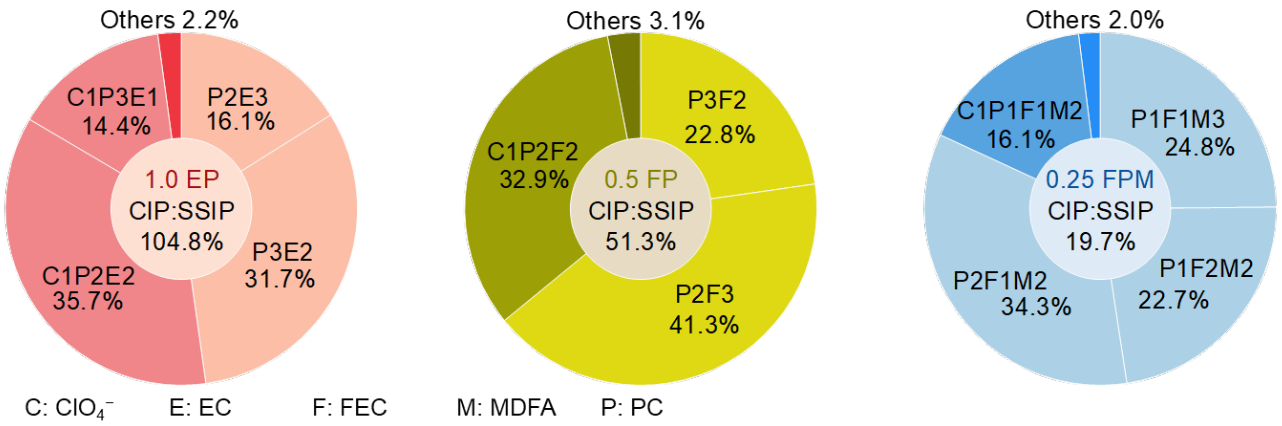

Figure S9. Solvation structure statistic and CIP:SSIP ratio of the 1.0 EP, 0.5 FP, and 0.25 FPM.

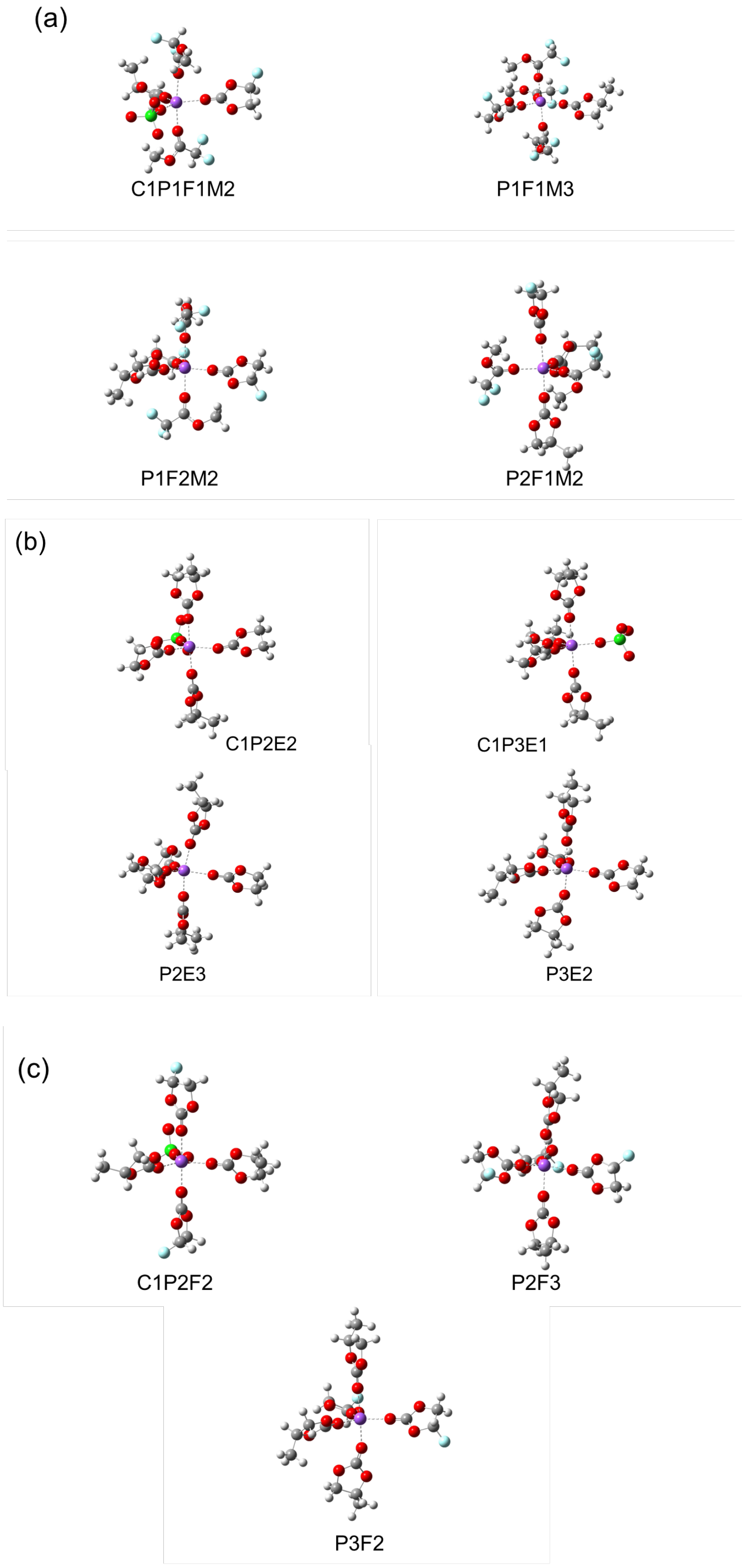

Figure S10. Solvation structure of (a) 0.25 FPM and (b) 1.0 EP and (c) 0.5 FP

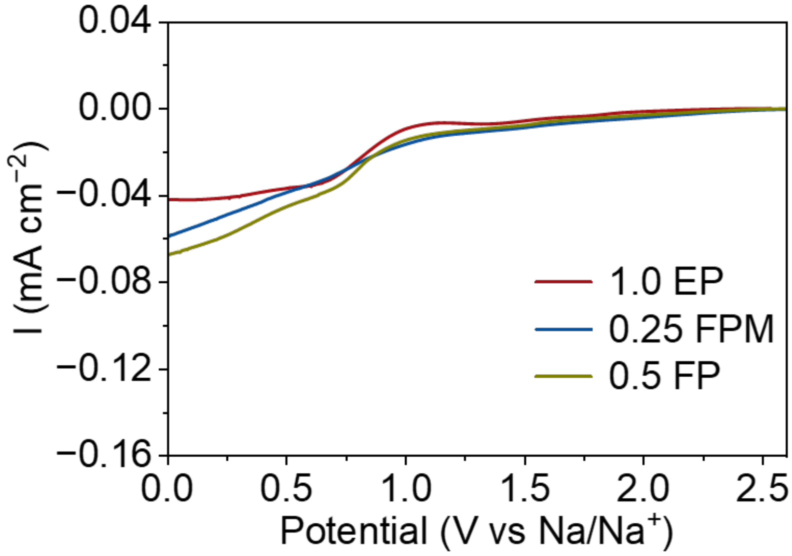

Figure S11. Cathodic limit of 1.0 EP, 0.5 FP and 0.25 FPM measured by LSV with 0.1 mV s^{-1}.

Although the anodic limits of these electrolytes are varied, the cathodic limits are lied in the reverse trend of the anodic limits.

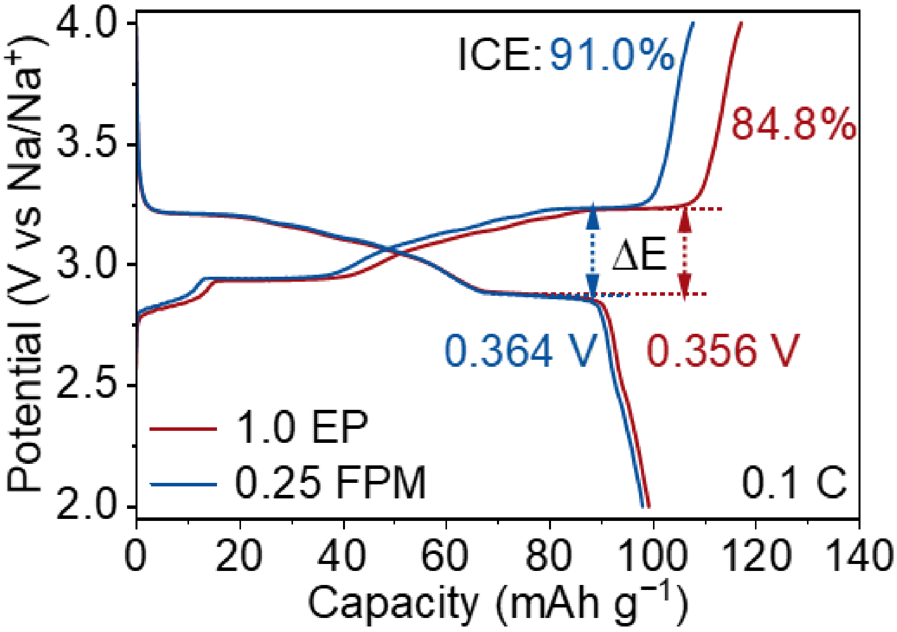

Figure S12. Initial GCD curve and ICE of NFPP with 1.0 EP and 0.25 FPM at 0.1 C.

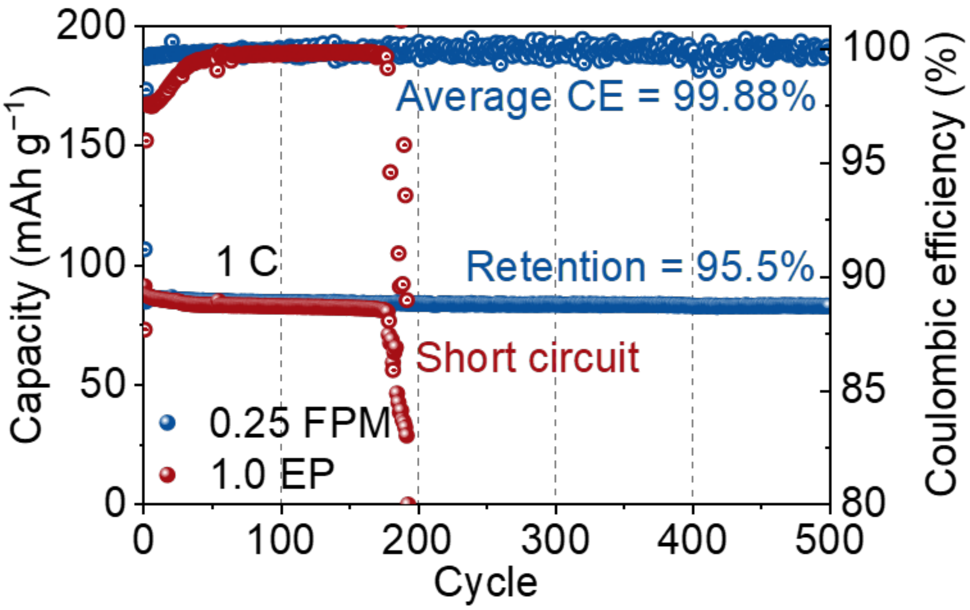

Figure S13. 1 C Cycling comparison between 1.0 EP and 0.25 FPM. 0.25 FPM presents good stability with high potential of 4 V vs Na/Na^{+}.

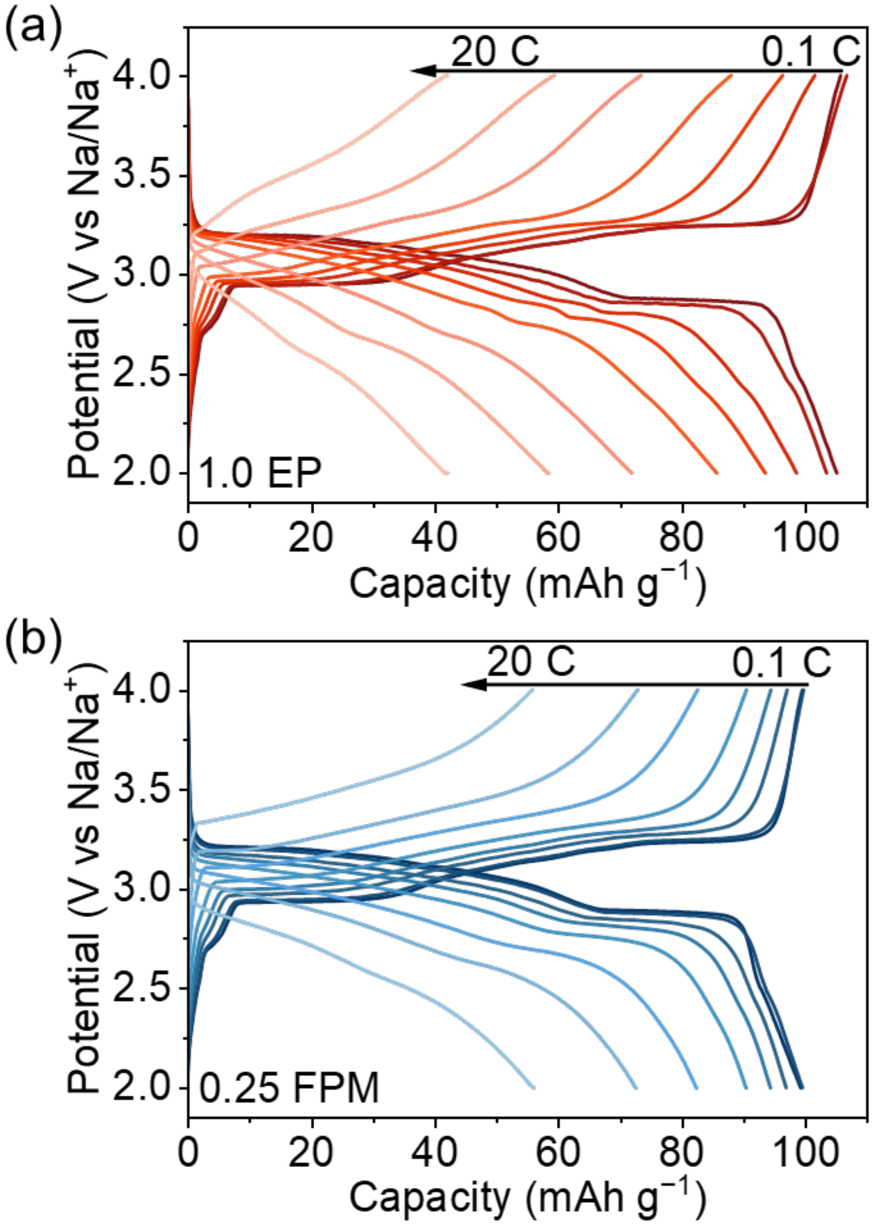

Figure S14. Charge and discharge curve of NFPP//Na half-cell at various rate with (a) 1.0 EP and (b) 0.25 FPM. Although larger initial polarization is observed in the GCD at higher rate, the platform stage of discharge curves are maintained well for 0.25 FPM.

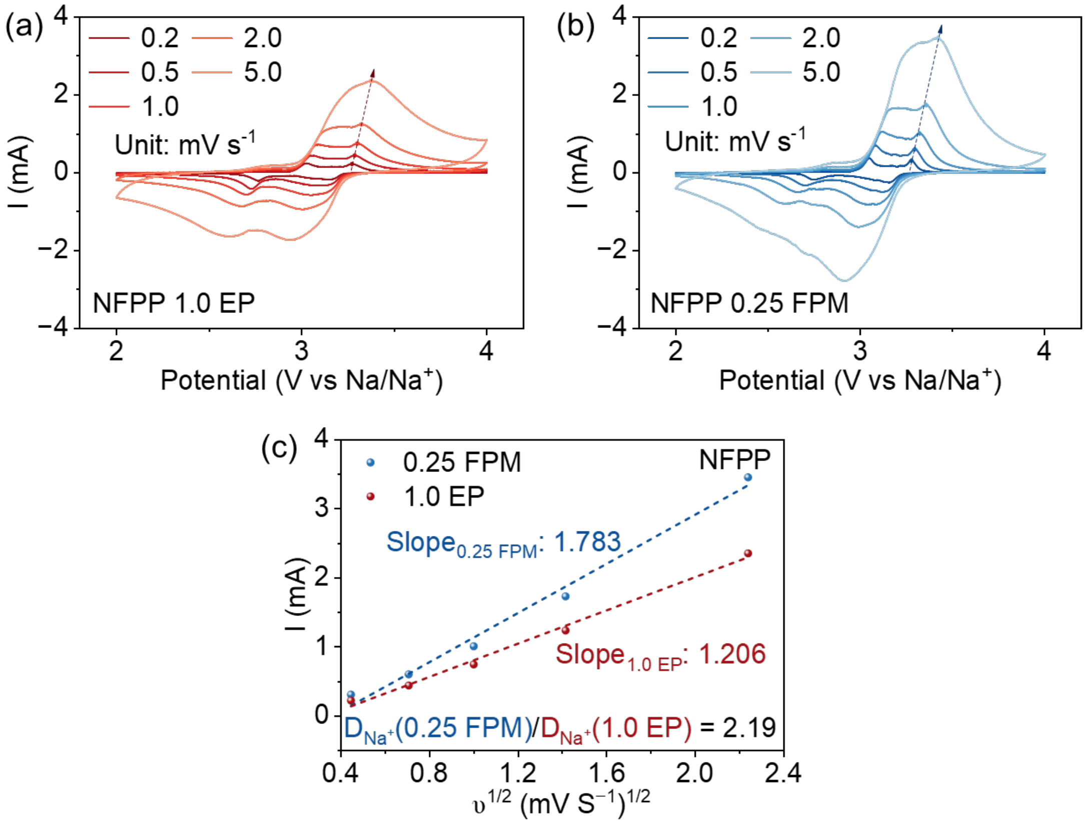

Figure S15. CV curve of NFPP//Na half-cell with (a) 1.0 EP and (b) 0.25 FPM. (c) Sodium ion diffusion ratio between 1.0 EP and 0.25 FPM in NFPP//Na.

Peak currents at anodic scans were recorded in each scan rate for further comparison of diffusion coefficients. Comparison of diffusion coefficients were carried out by using Randles-Sevcik equation:

$$I_p = 0.446nFAC(nF\upsilon D_{Na}/RT)^{0.5} \quad (4)$$

where Ip is the peak current at each scan rate, n is the electron transferred in redox reactions, F is the Faradic constant, A is the surface area of the electrode, C is the bulk concentration, υ is the scan rate and D_{Na} is the sodium ion diffusion coefficient. Therefore, the D_{Na} ratio between electrolytes can be evaluated from:

$$D_{Na(1)}/D_{Na(2)} = (I_{p1}^2/\upsilon)/(I_{p2}^2/\upsilon) = (Slope_1/Slope_2)^2 \quad (5)$$

Noting this diffusion coefficient represent the bulk diffusion coefficient inside the cell, the low viscosity of 0.25 FPM has benefitted this process.

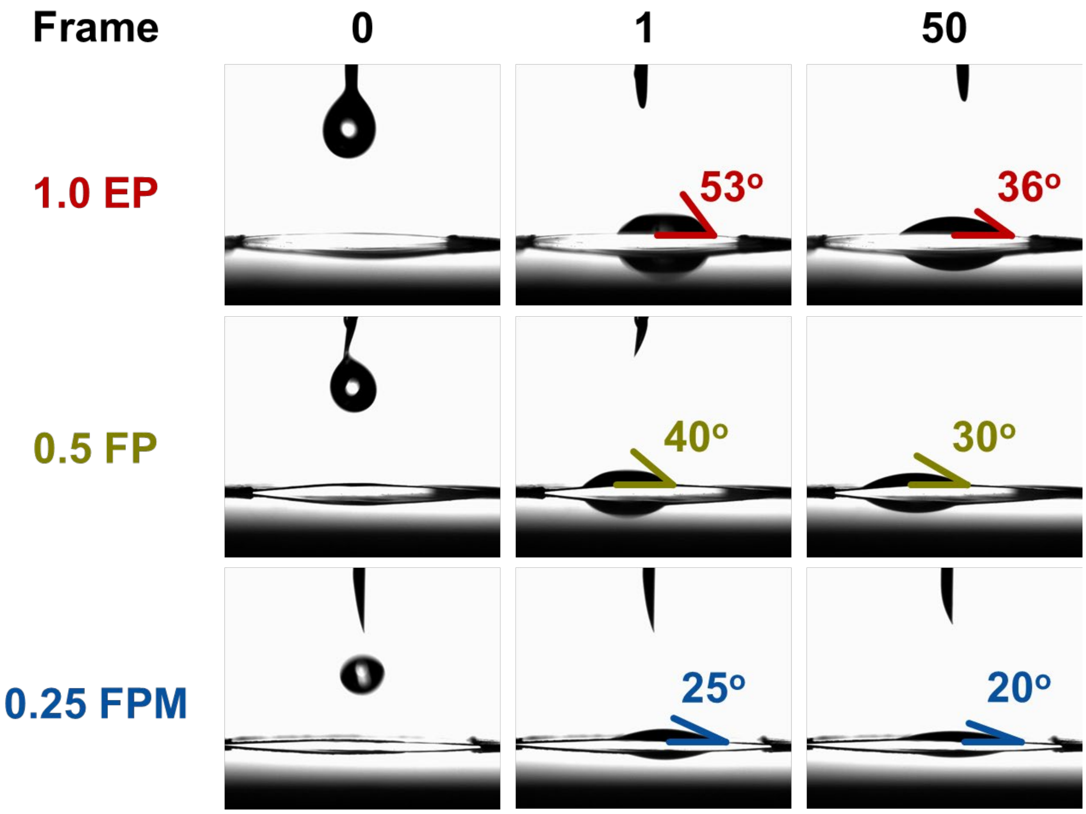

Figure S16. Contact angle test of 1.0 EP, 0.5 FP and 0.25 FPM with NFPP cathode film.

The records were starting at the drop falling, the contact angles were measured at instant contact and 50 frames later.

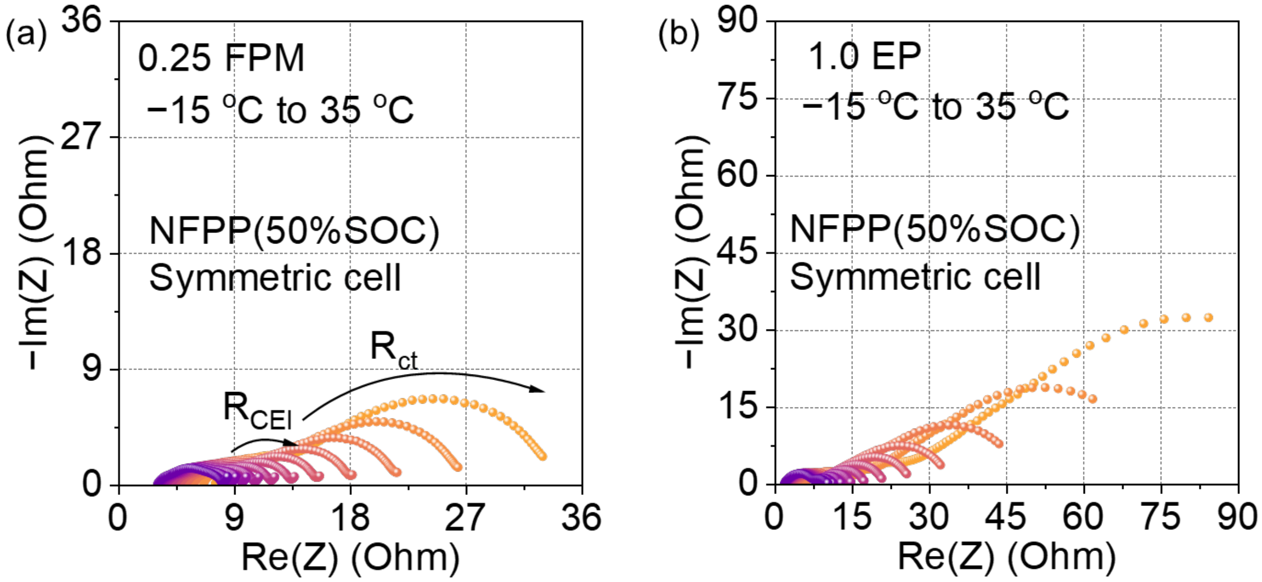

Figure S17. Temperature dependent Nyquist plot of NFPP symmetric cell of (a) 0.25 FPM and (b) 1.0 EP.

Half cells with 0.25 FPM and 1.0 EP were first cycled at 2-4 V, 0.1 C for 5 cycles to create stable CEI. During the sixth charge, the battery was charged to 3.1 V by constant current and followed by constant voltage charge until the current reach 0.01 C to keep the cathode at constant state, a pair of electrodes with similar active material mass were assembled for EIS measuring. R_{CEI} is used to evaluate CEI diffusion E_a and R_{ct} is used to evaluate de-solvation E_a.

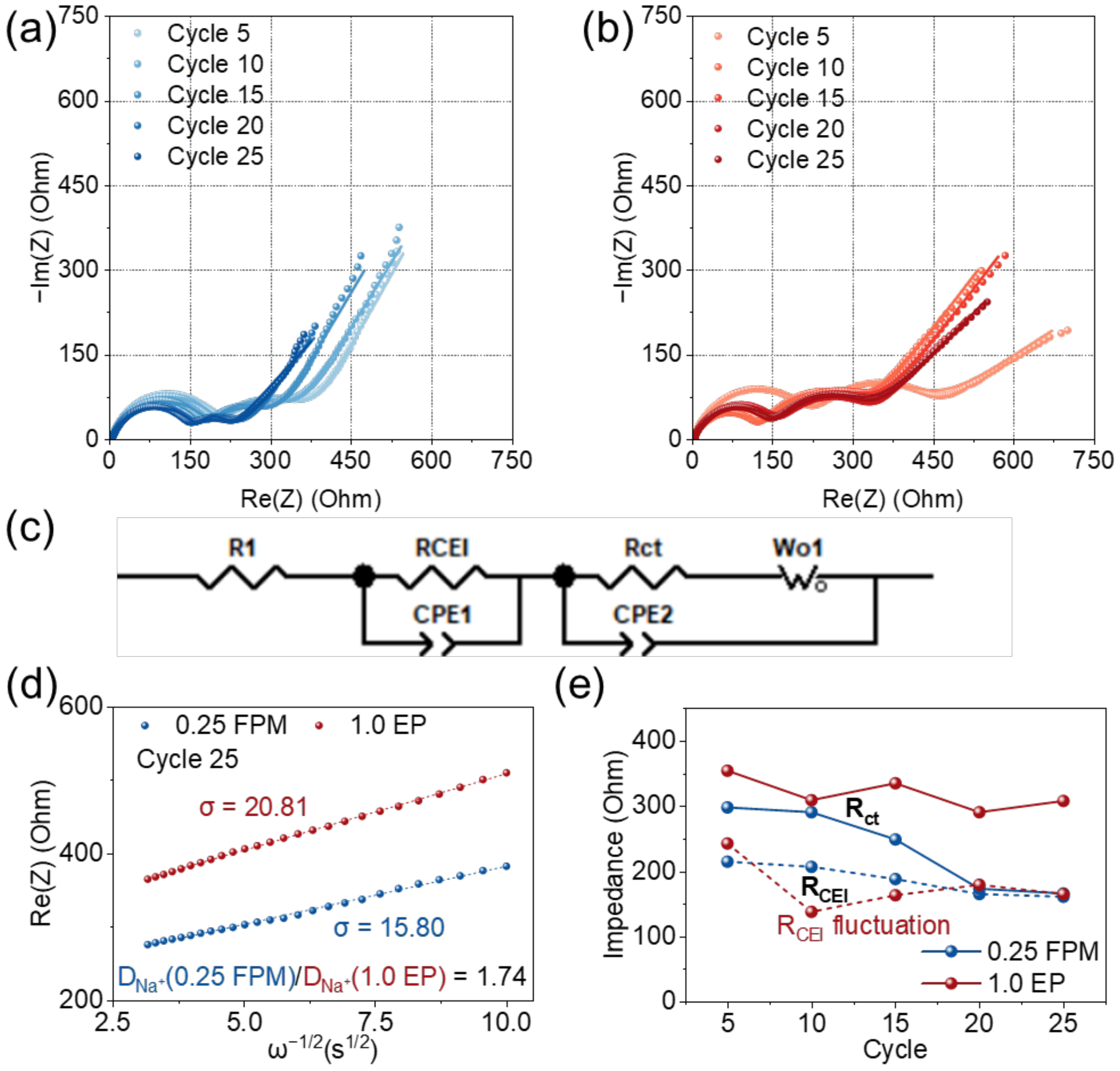

Figure S18. Nyquist plot and fitting lines of NFPP//Na half-cells at cycle 5 to cycle 25 with (a) 0.25 FPM, (b) 1.0 EP, and (c) equivalent circuit. (d) R_{CEI} and R_{ct} fitted from Nyquist plot of NFPP half cell at the 5th cycle to 25th cycle with 0.25 FPM and 1.0 EP. (e) Diffusion coefficient ratio fitted from low frequency region at cycle 25.

Warburg factor σ is the slope of Z_{real} vs $\omega^{-1/2}$ in the low frequency zone. According to equation (6):

$$D_{Na} = (RT)^2/2A^2F^4n^4C^2\sigma^2 \quad (6)$$

Where R is the gas constant, T is the room temperature, A is the surface area of the NFPP cathode, F is the Faraday constant, n is the electron number, C is the concentration of the sodium ion, and σ is the Warburg factor. Therefore, the D_{Na} ratio between electrolytes can be evaluated from:

$$D_{Na(1)}/D_{Na(2)} = (\sigma_2/\sigma_1)^2 \quad (7)$$

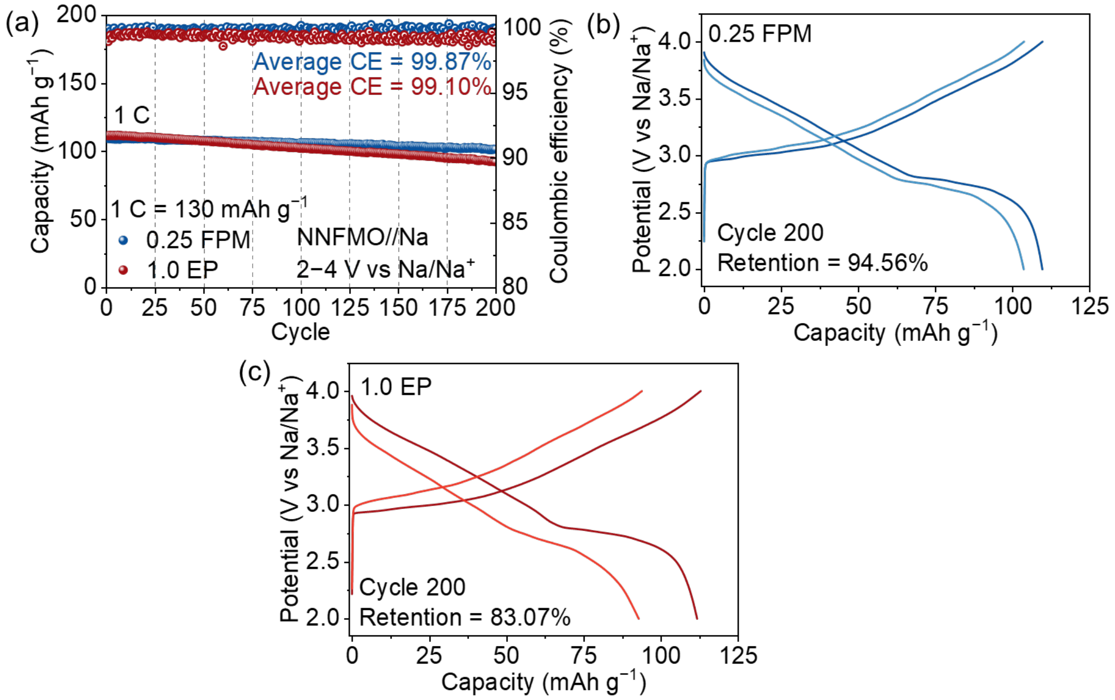

Figure S19. (a) 1 C cycling test of $Na(Ni_{1/3}Fe_{1/3}Mn_{1/3})O_2$ (NNFMO)//Na half-cell with 0.25 FPM and 1.0 EP. Charge and discharge curve of cycle 1 and cycle 200 of NNFMO//Na half-cell with (b) 0.25 FPM and (c) 1.0 EP.

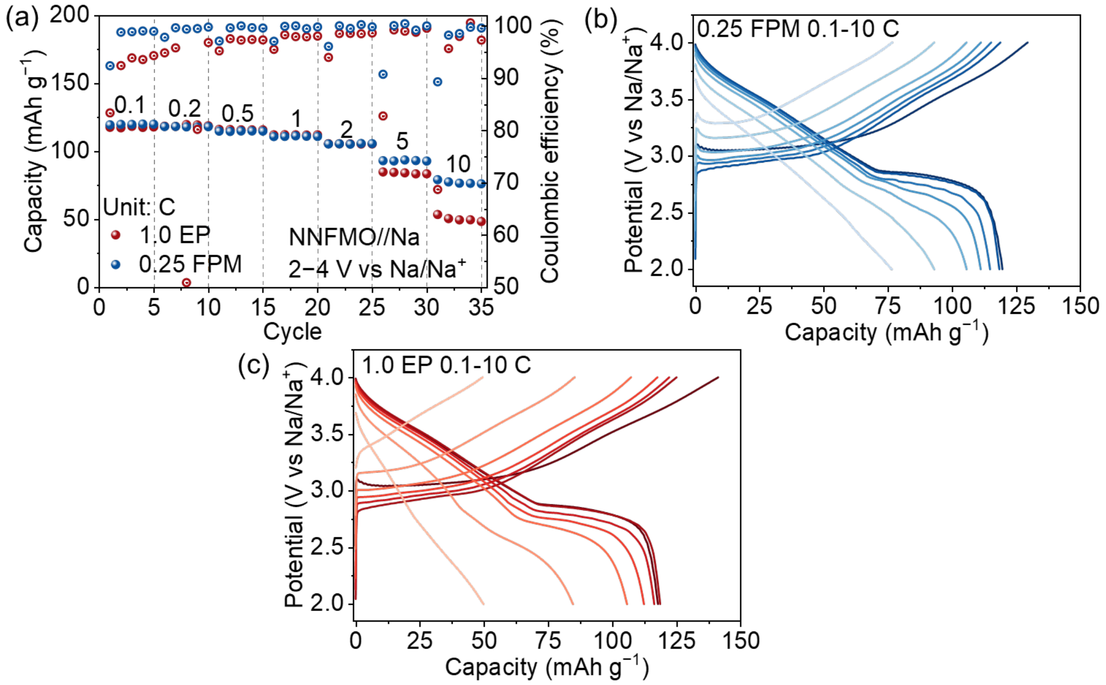

Figure S20. (a) Rate performance of NNFMO//Na half-cell with 0.25 FPM and 1.0 EP. Charge and discharge curve of NNFMO//Na half-cell with (b) 0.25 FPM and (c) 1.0 EP at each rate.

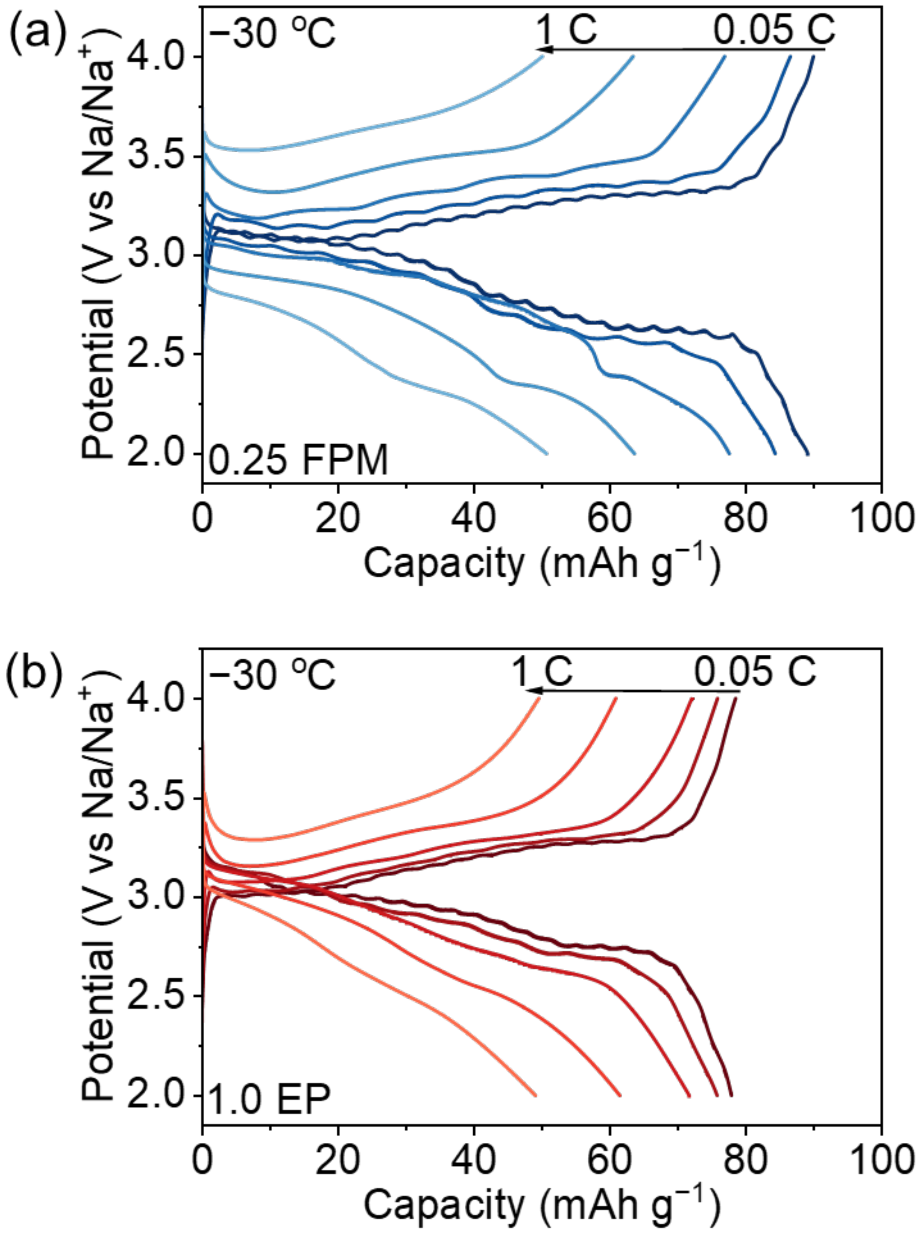

Figure S21. Charge and discharge curve of NFPP//Na half-cell with (a) 0.25 FPM and (b) 1.0 EP from 0.05 C to 1 C at −30 °C.

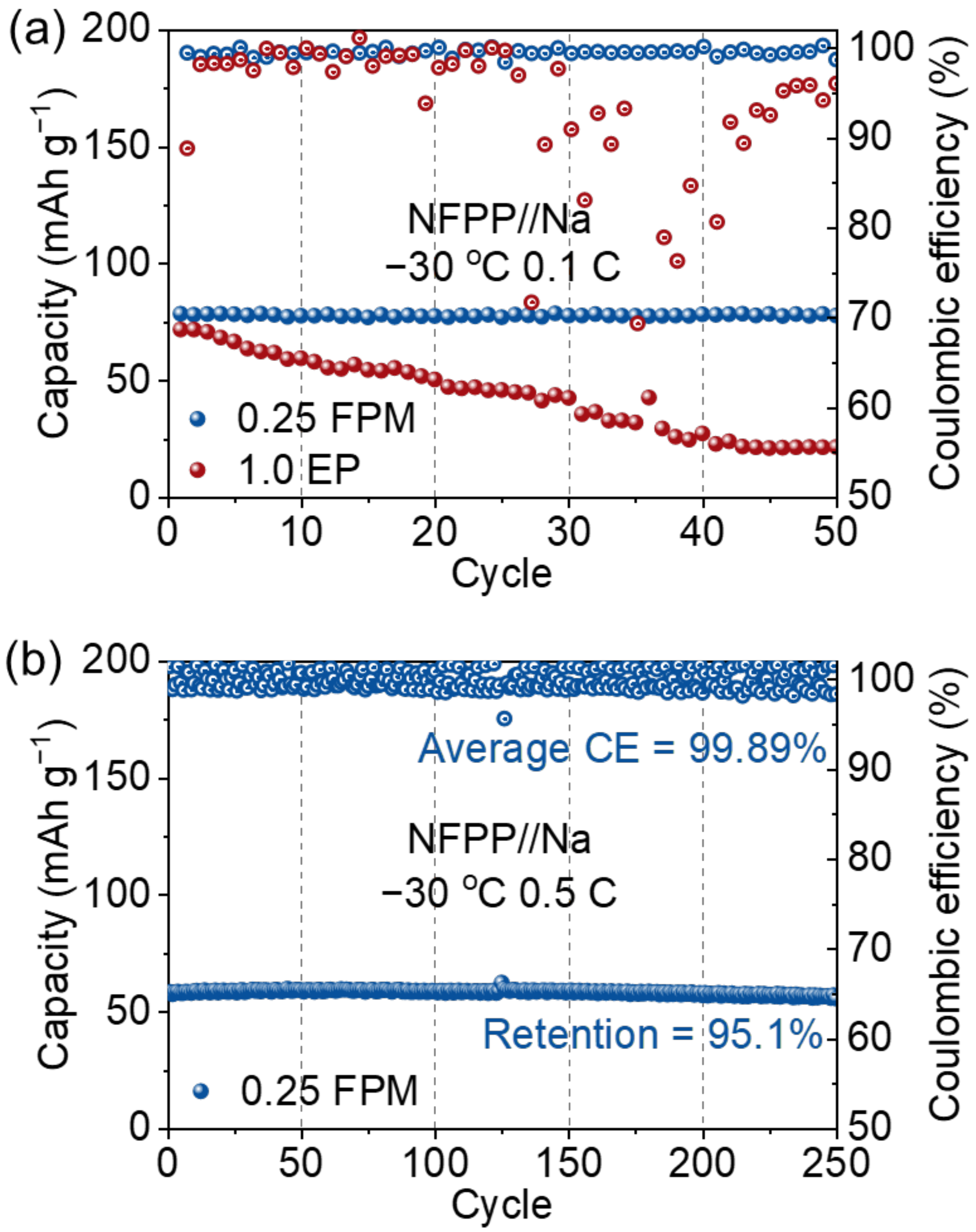

Figure S22. (a) Comparison of cycling stability of NFPP//Na half-cell with 0.25 FPM and 1.0 EP at 0.1 C and −30 °C. (b) Cycling performance of NFPP//Na half-cell with 0.25 FPM at 0.5 C and −30 °C.

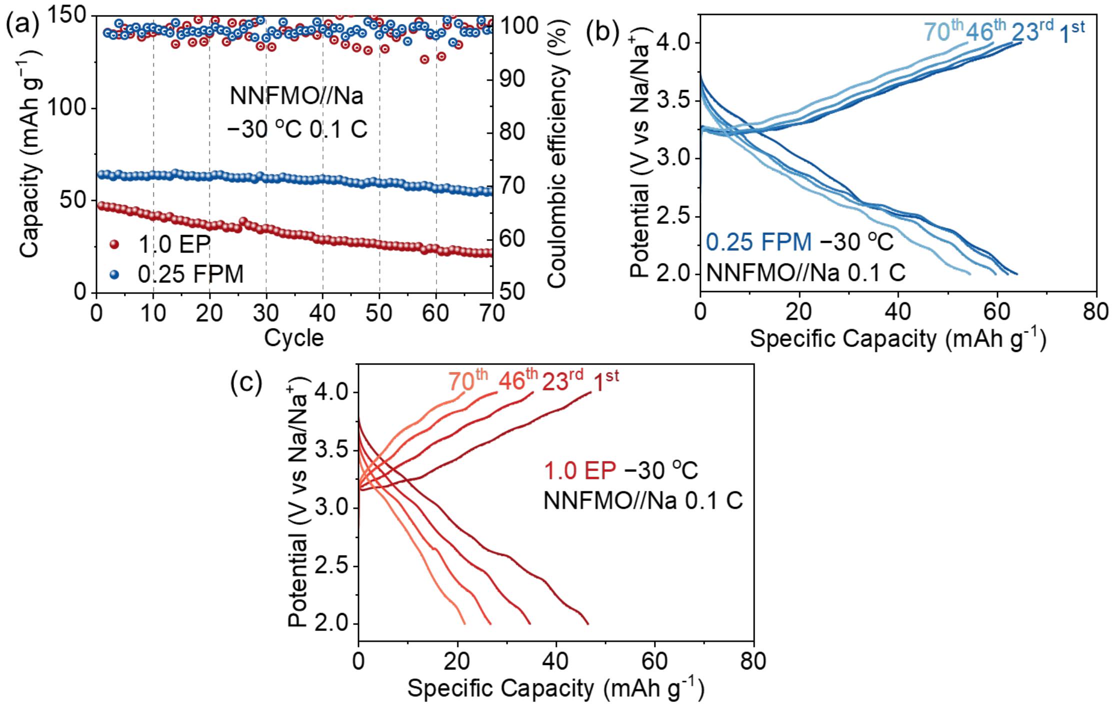

Figure S23. (a) Comparison of cycling stability of NNFMO||Na half-cell with 0.25 FPM and 1.0 EP at 0.1 C, −30 °C. The 1st, 23rd, 46th and 70th charge and discharge curves of NNFMO||Na half-cell with (b) 0.25 FPM and (c) 1.0 EP at 0.1 C, −30 °C.

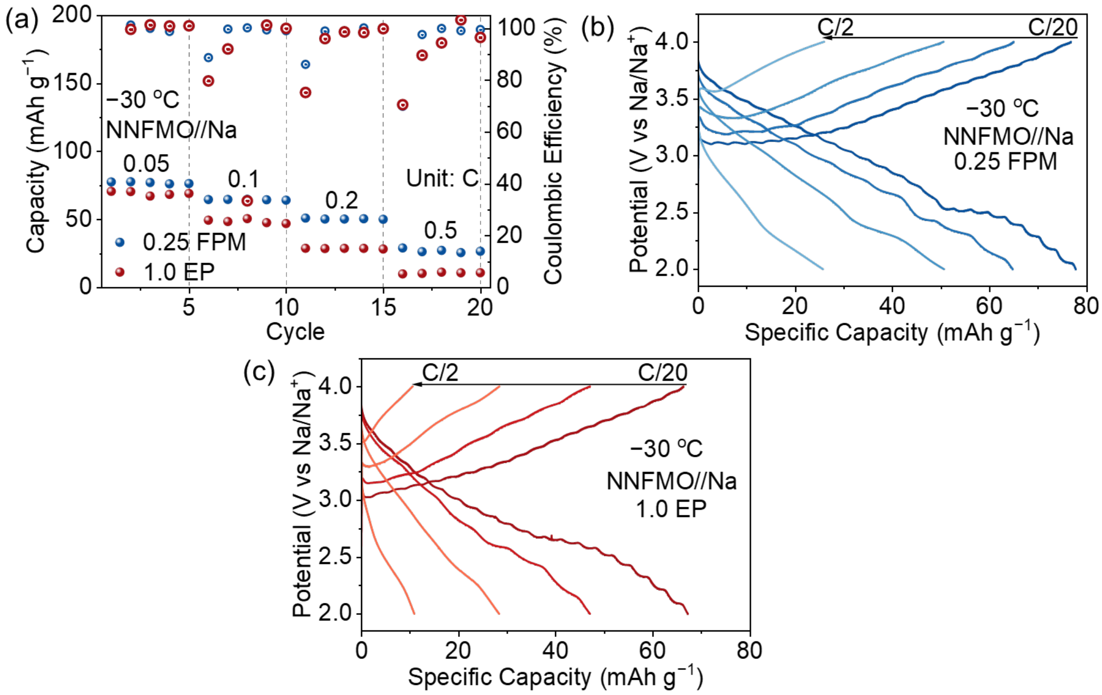

Figure S24. (a) Rate performance of NNFMO||Na half-cell with 0.25 FPM and 1.0 EP from 0.05 C to 0.5 C. Charge and discharge curve of NNFMO||Na half-cell with (b) 0.25 FPM and (c) 1.0 EP at each rate.

Owing to the structural differences between layered NNFMO and 3D channel NFPP, NFPP has a relatively better low-temperature performance. While the 0.25 FPM still perform well with NNFMO cathode.

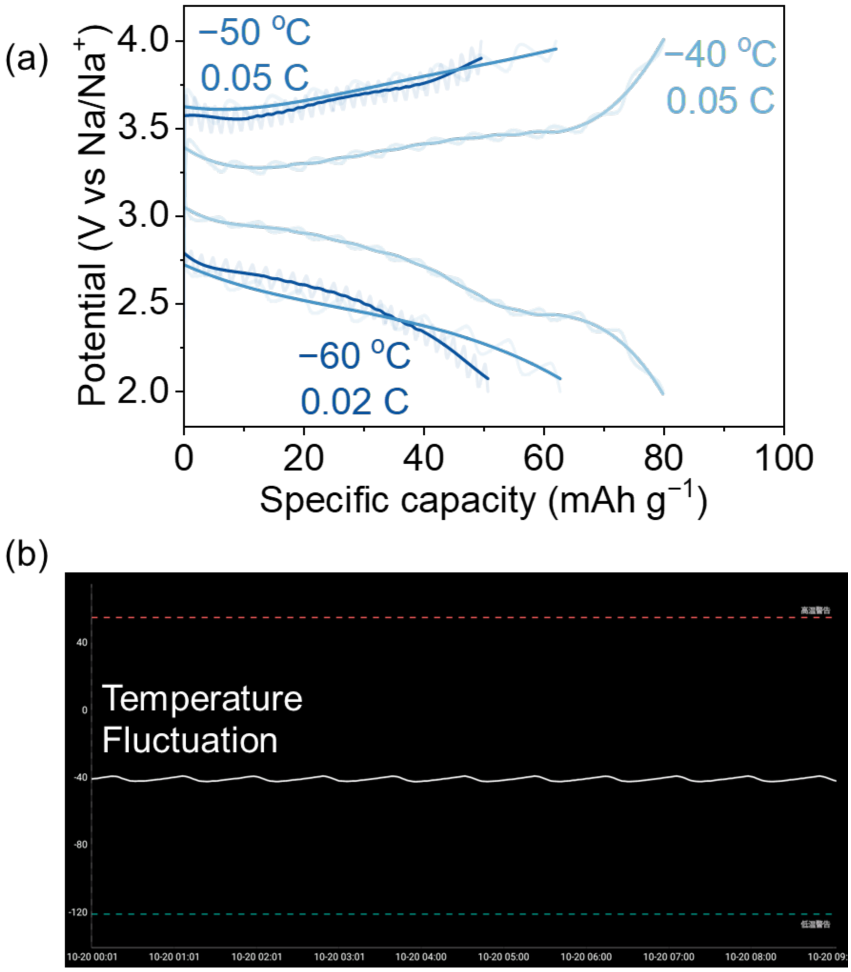

Figure S25. (a) Charge and discharge curves of NFPP||Na half-cell with 0.25 FPM at 0.05 C, −40 °C, −50 °C and 0.02 C, −60 °C, (b) Time dependent temperature fluctuation of the low low-temperature testing box.

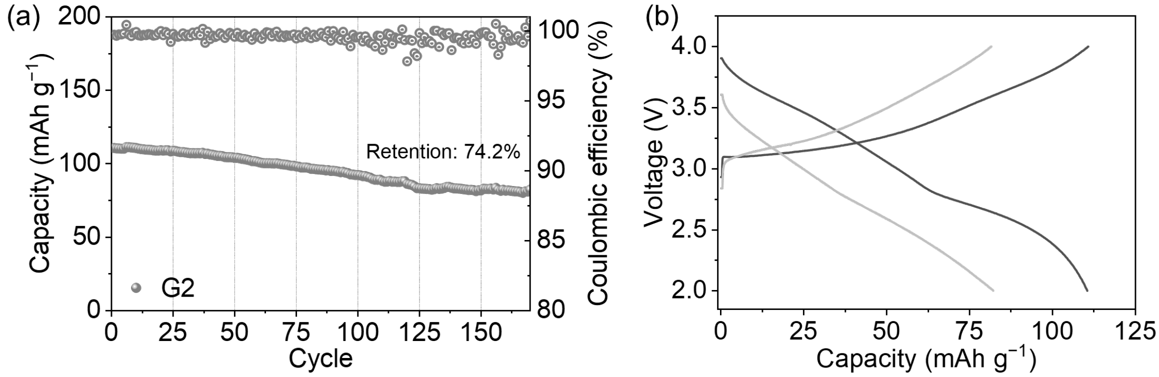

Figure S26. (a) 1 C cycling test of $Na(Ni_{1/3}Fe_{1/3}Mn_{1/3})O_2$ (NNFMO)//Na half-cell with 1.0 M $NaPF_6$ DEGDME electrolyte (b) Charge and discharge curve of cycle 1 and cycle 170 of NNFMO//Na half-cell.

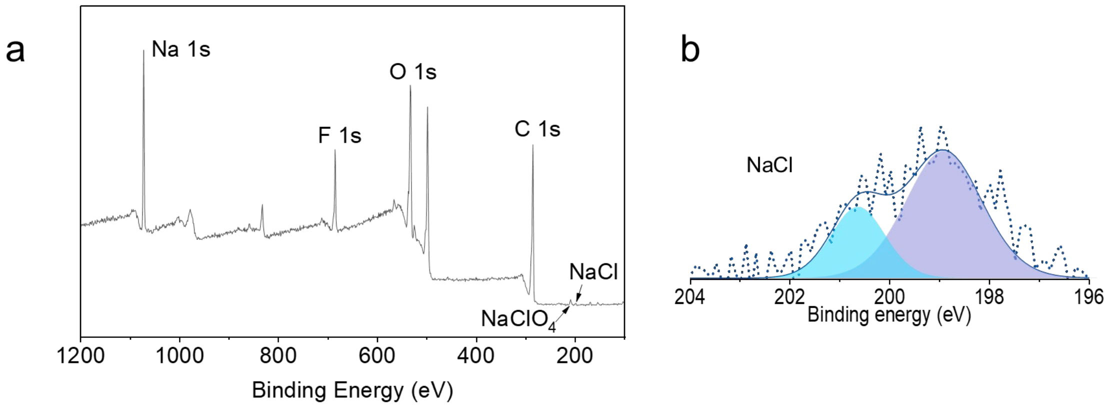

Figure S27. (a) Full spectrum and (b) Cl 2p of the XPS results on the hard carbons surface of 0.25 FPM.

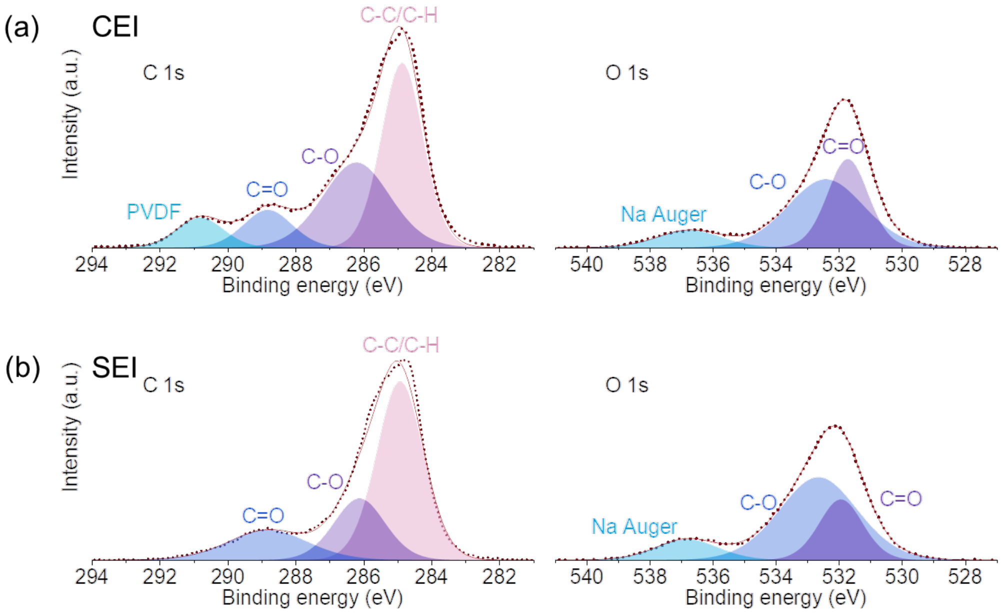

Figure S28. C 1s and O 1s XPS spectra of (a) CEI and (b) SEI in NFPP||HC full-cell with 1.0 EP.

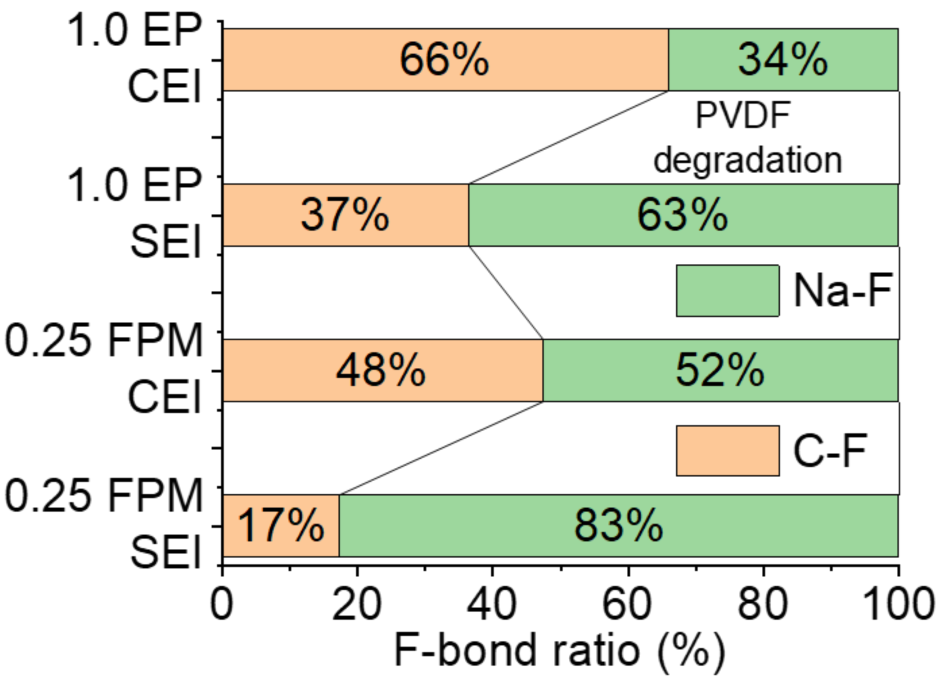

Figure S29. C-F and Na-F ratio comparison in the CEI and SEI of 0.25 FPM and 1.0 EP.

In 1.0 EP, the only F source is the PVDF used in the fabrication of cathode slurry. Reports has been shown that the degradation of PVDF can be the source of NaF in batteries. Although in the SEI of 1.0 EP there are 63% Na-F bond, the total F atom ratio was only 3%, it is highly possibly caused by the dissolved NaF in EC:PC.

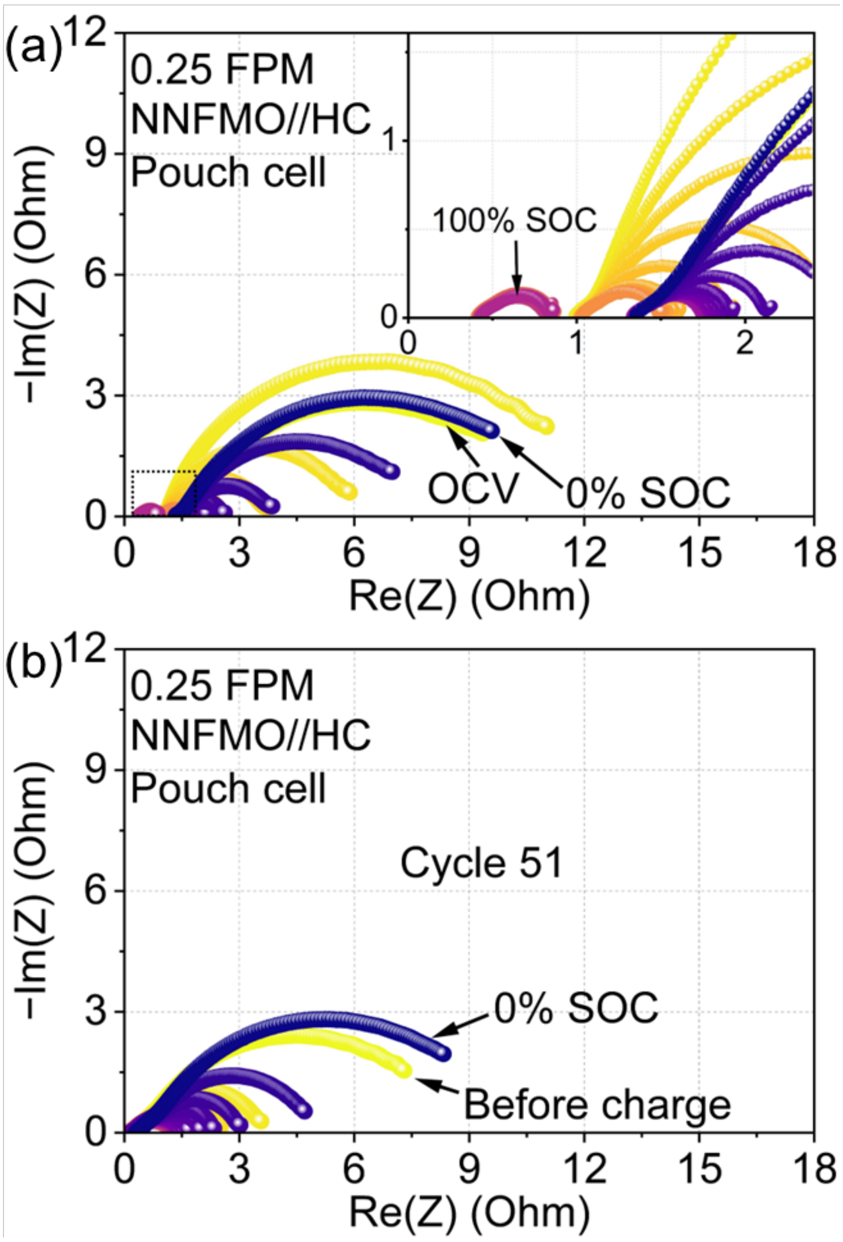

Figure S30. Nyquist plots of the In-situ EIS at (a) cycle 1 and (b) cycle 51.

For such low impedance and overlapped Nyquist plots, traditional fitting is not precise enough to distinguish the evolution of impedance upon cycling.

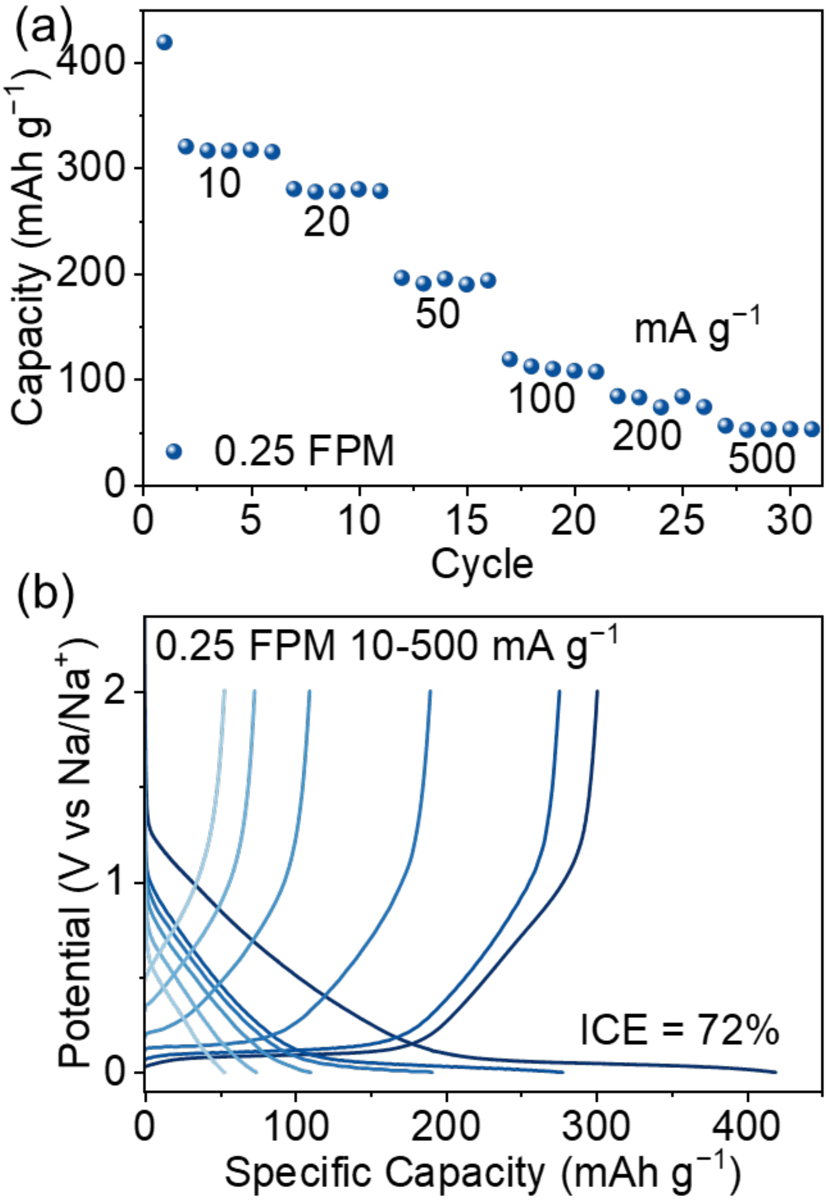

Figure S31. (a) Discharge capacity and (b) GCD curve of HC||Na half-cell with 0.25 FPM electrolyte at different current densities.

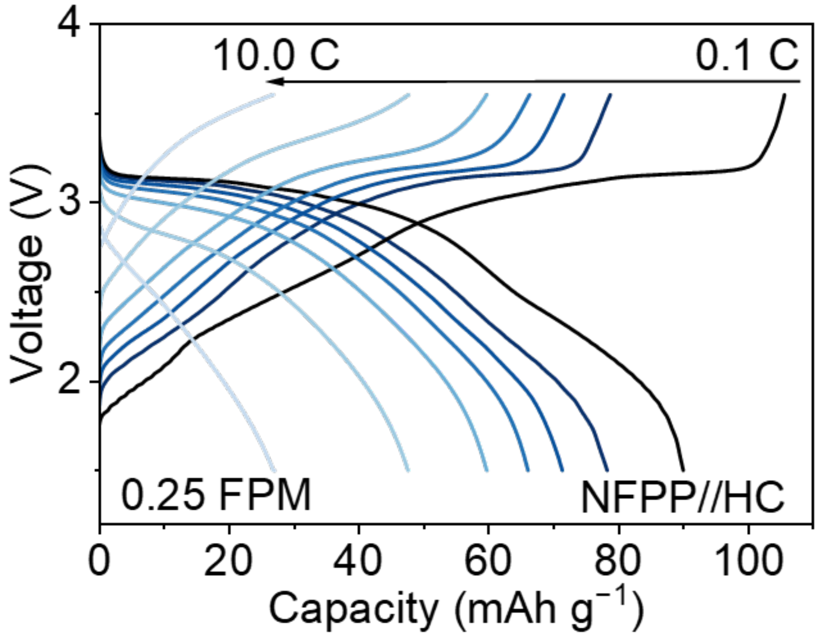

Figure S32. GCD curve of $Na_4Fe_3(PO_4)_2P_2O_7$||HC full-cell with 0.25 FPM electrolyte from 0.1 C to 10 C.

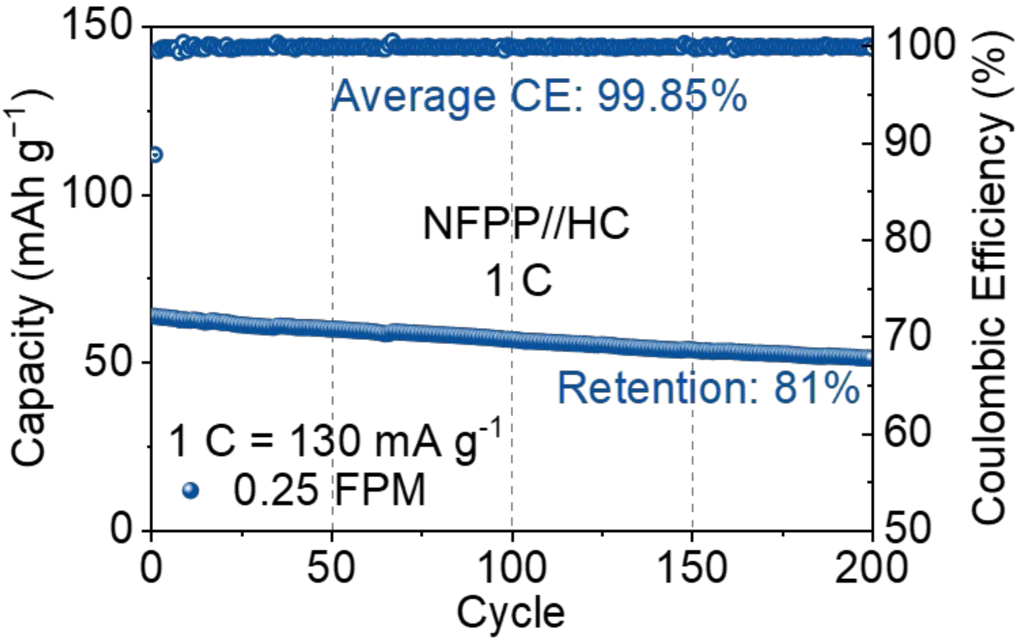

Figure S33. 1 C cycling test of NFPP||HC coin cell with 0.25 FPM.

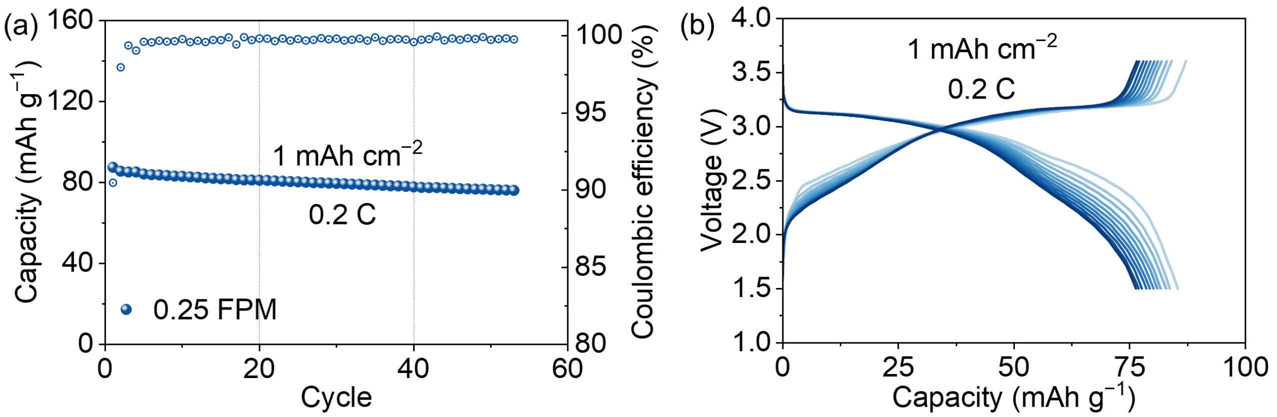

Figure S34. (a) Cycling retention and coulombic efficiency and (b) Charge and Discharge curves of NFPP||HC full cell with areal loading of 1 mAh cm^{-2}.

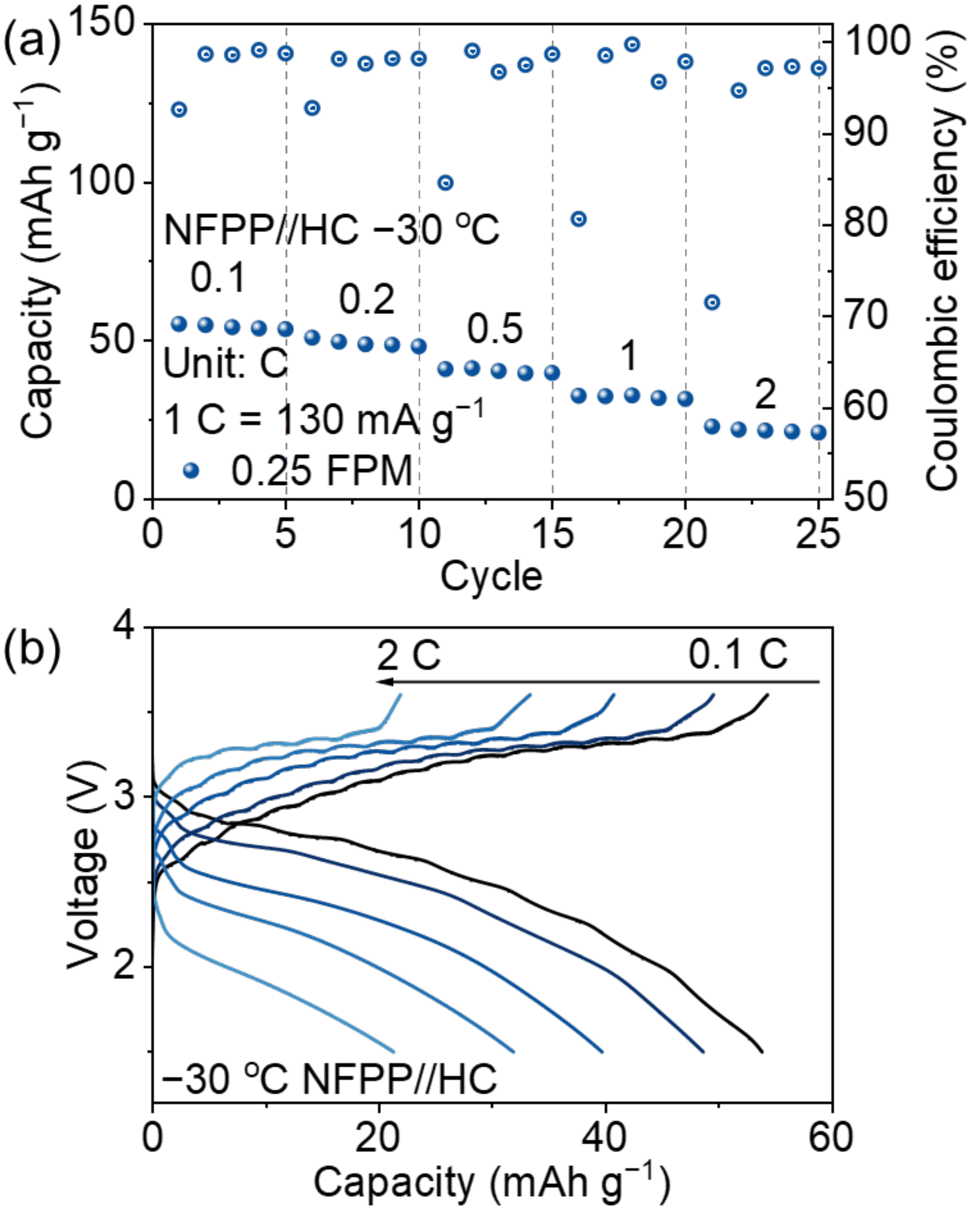

Figure S35. (a) Rate performance and (b) GCD curves of NFPP||HC coin cell with 0.25 FPM electrolyte from 0.1 C to 2 C at −30 °C.

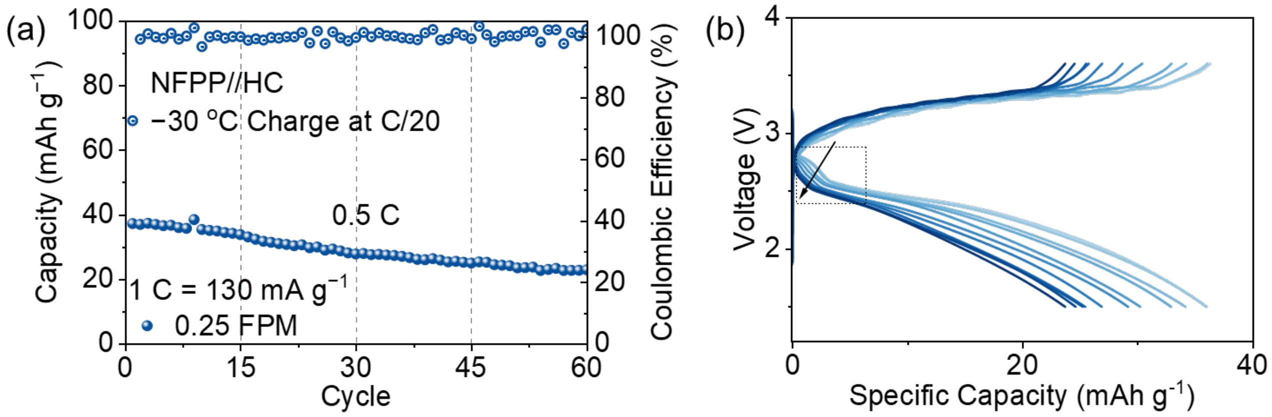

Figure S36. (a) Discharge capacity, coulombic efficiency, (b) GCD curve of NFPP||HC coin cell at −30 °C 0.5 C.

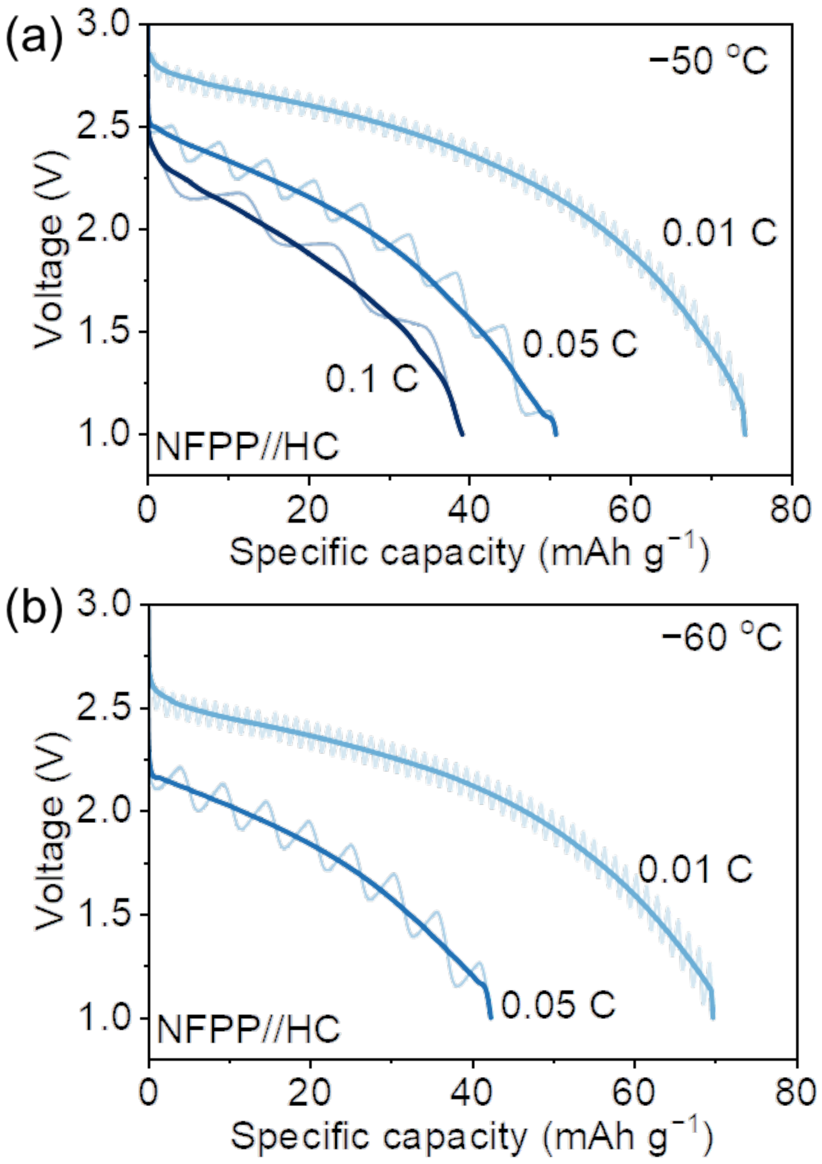

Figure S37. Discharge capacities and curves of NFPP||HC full-cell at (a) −50 °C, 0.01, 0.05 and 0.1 C, (b) −60 °C, 0.01 and 0.05 C,

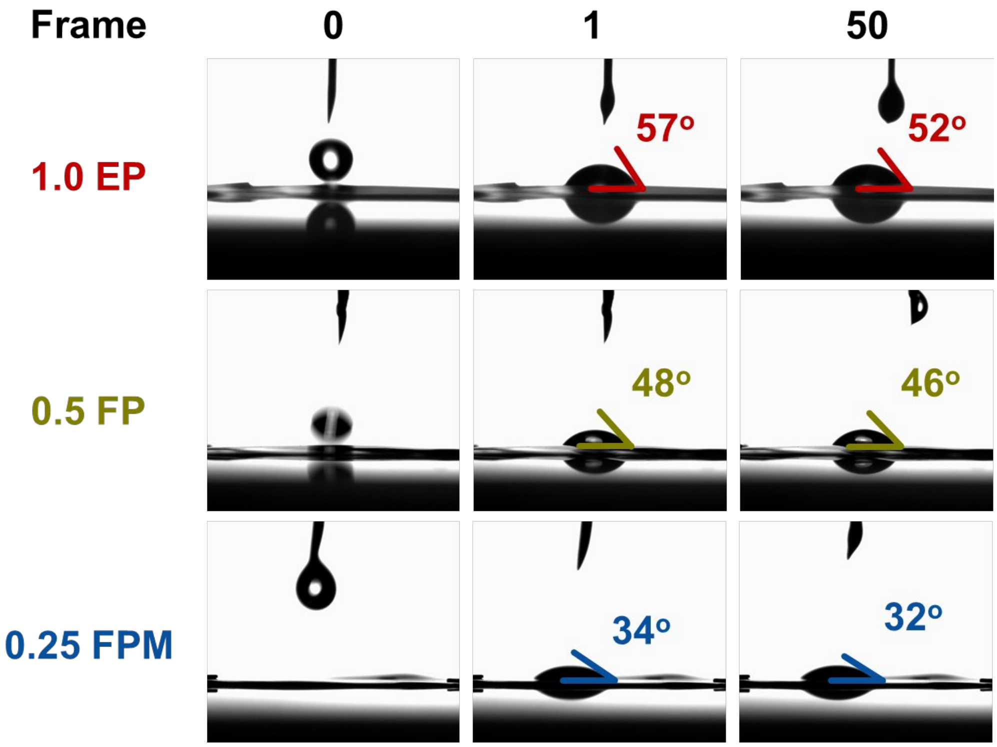

Figure S38. Contact angle test of 1.0 EP, 0.5 FP and 0.25 FPM with Celgard 2325 separator.

The records were starting at the drop falling, the contact angles were measured at instant contact and 50 frames later.

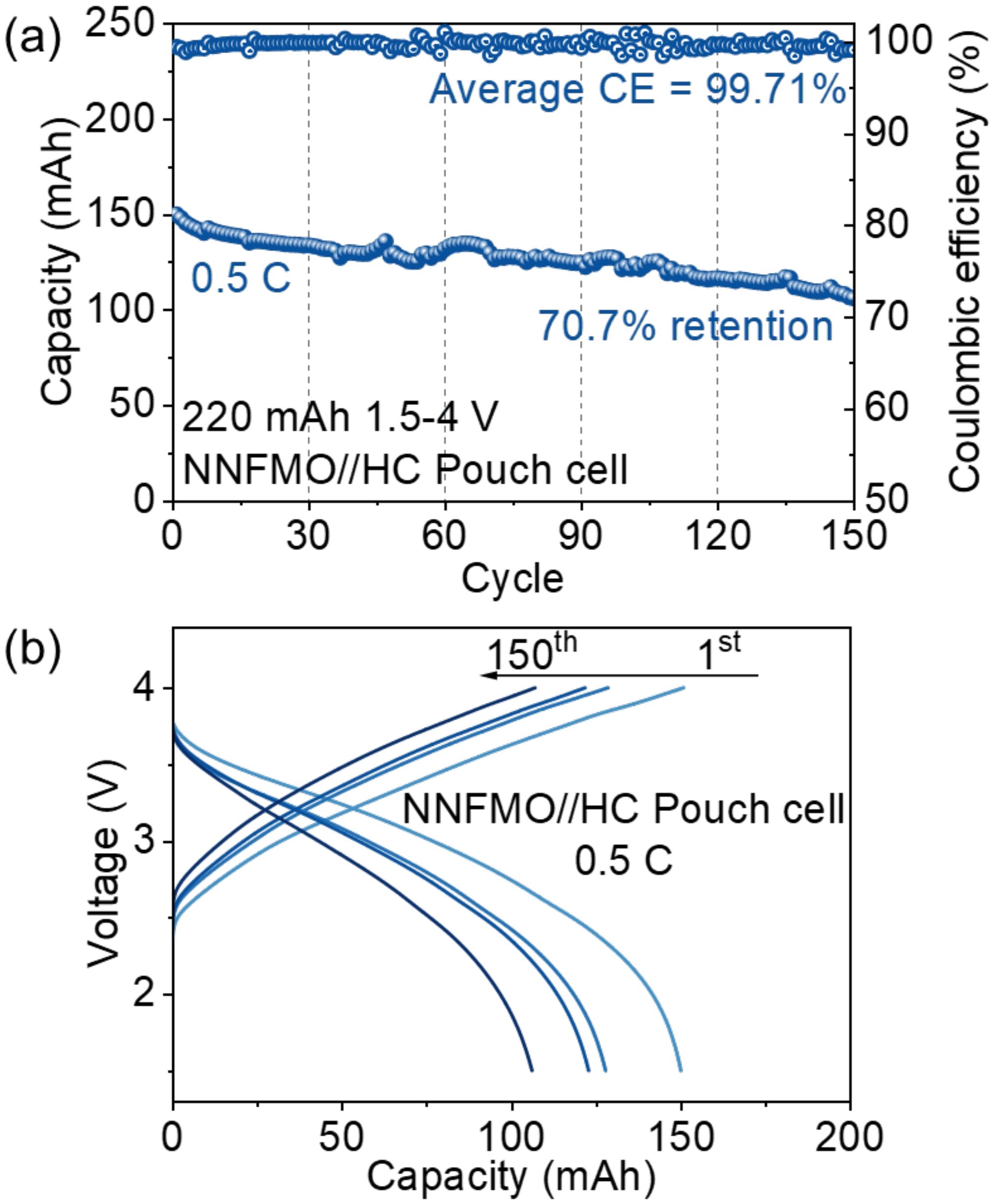

Figure S39. (a) Cycling stability of NNFMO||HC pouch cell at 0.5 C. (b) Charge and discharge curve of the 1st, 50th, 100th, and 150th cycle.

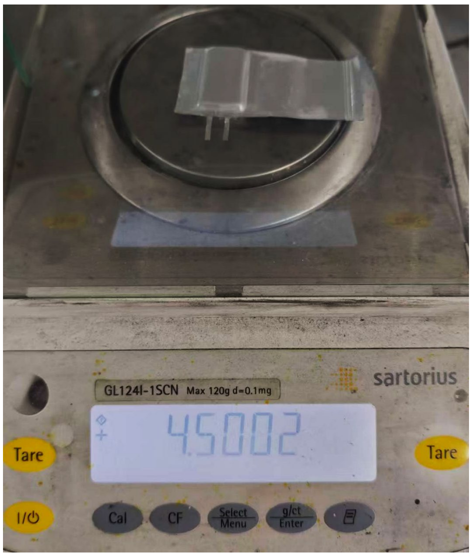

Figure S40. Mass of a fresh commercial NNFMO||HC pouch cell

Table S1. Technical parameter of NNFMO||HC pouch cell

Typical Capacity	220 mAh
Cell Mass	4.5 g
Electrolyte Usage	1.1 g
Voltage	1.5-4.0 V
Initial discharge capacity (energy)	184 mAh (564 mWh)
25 °C Energy Density	100.7 Wh kg^{-1}
Discharge capacity (energy)	145 mAh (354 mWh)
−30 °C Energy Density	63.2 Wh kg^{-1}

Table S2. Discharge capacity retention at low temperature of pouch cell from literature

Retention	Paper	Reference
−20 °C, 75.3%	Adv. Mater. 2024	[4]
−25 °C, 87.7%; −40 °C, 56%	J. Am. Chem. Soc. 2025	[5]
−20 °C, 79.3%	Chem. Engin. J. 2025	[6]
−30 °C, 73.3%	Adv. Energy Mater 2025	[7]
−40 °C, 85.8%	Adv. Energy Mater 2025	[8]

Reference

1. Wang Y-L, Shah FU, Glavatskih S *et al*. Atomistic insight into orthoborate-based ionic liquids: Force field development and evaluation. *J Phys Chem B* 2014; **118**: 8711-23.

2. Doherty B, Zhong X, Gathiaka S *et al*. Revisiting opls force field parameters for ionic liquid simulations. *J Chem Theory Comput* 2017; **13**: 6131-45.

3. Abraham MJ, Murtola T, Schulz R *et al*. Gromacs: High performance molecular simulations through multi-level parallelism from laptops to supercomputers. *SoftwareX* 2015; **1-2**: 19-25.

4. Liao Y, Yuan L, Han Y *et al*. Pentafluoro(phenoxy)cyclotriphosphazene stabilizes electrode/electrolyte interfaces for sodium-ion pouch cells of 145 Wh kg^{-1}. *Adv Mater* 2024; **36**: 2312287.

5. Liang H-J, Qian W-Y, Liu H-H *et al*. Sulfite-based electrolyte chemistry with ion–dipole interactions and robust interphase achieves wide-temperature sodium-ion batteries. *Journal of the American Chemical Society* 2025; **147**: 17860-70.

6. Hu R, Yang L, Zhang C *et al*. Bifunctional sodium tetrakis [3,5-bis(trifluoromethyl)phenyl] borate additive for long-lifespan sodium-ion batteries with $NaNi_{0.33}Fe_{0.33}Mn_{0.33}O_2$ cathode. *Chem Eng J* 2025; **512**: 162144.

7. Cui Y, Ni Y, Wang Y *et al*. A temperature-adapted ultraweakly solvating electrolyte for cold-resistant sodium-ion batteries. *Adv Energy Mater* 2025; **15**: 2405363.

8. Xia M, Chen H, Zheng Z *et al*. Sodium-difluoro(oxalato)borate-based electrolytes for long-term cycle life and enhanced low-temperature sodium-ion batteries. *Adv Energy Mater* 2025; **15**: 2403306.
